# Single-cell RNA-seq integrated with multi-omics reveals SERPINE2 as a target for metastasis in advanced renal cell carcinoma

**DOI:** 10.1038/s41419-023-05566-w

**Published:** 2023-01-16

**Authors:** Wen-jin Chen, Ke-qin Dong, Xiu-wu Pan, Si-shun Gan, Da Xu, Jia-xin Chen, Wei-jie Chen, Wen-yan Li, Yu-qi Wang, Wang Zhou, Brian Rini, Xin-gang Cui

**Affiliations:** 1 Department of Urology, Xinhua Hospital, School of Medicine, Shanghai Jiaotong University, 1665 Kongjiang Road, Shanghai, 200092 China; 2 Department of Urology, Third Affiliated Hospital of the Second Military Medical University, Shanghai, 201805 China; 3 Department of Urology, General Hospital of Central Theater Command of PLA, Wuhan, 430070 China; 4 Division of Hematology Oncology, Vanderbilt University Medical Center, Nashville, TN USA

**Keywords:** Prognostic markers, Renal cell carcinoma, Molecular biology, Cancer genomics

## Abstract

Tumor growth, metastasis and therapeutic response are believed to be regulated by the tumor and its microenvironment (TME) in advanced renal cell carcinoma (RCC). However, the mechanisms underlying genomic, transcriptomic and epigenetic alternations in RCC progression have not been completely defined. In this study, single-cell RNA-sequencing (scRNA-seq) data were obtained from eight tissue samples of RCC patients, including two matched pairs of primary and metastatic sites (lymph nodes), along with Hi-C, transposable accessible chromatin by high-throughput (ATAC-seq) and RNA-sequencing (RNA-seq) between RCC (Caki-1) and human renal tubular epithelial cell line (HK-2). The identified target was verified in clinical tissue samples (microarray of 407 RCC patients, TMA-30 and TMA-2020), whose function was further validated by in vitro and in vivo experiments through knockdown or overexpression. We profiled transcriptomes of 30514 malignant cells, and 14762 non-malignant cells. Comprehensive multi-omics analysis revealed that malignant cells and TME played a key role in RCC. The expression programs of stromal cells and immune cells were consistent among the samples, whereas malignant cells expressed distinct programs associated with hypoxia, cell cycle, epithelial differentiation, and two different metastasis patterns. Comparison of the hierarchical structure showed that SERPINE2 was related to these NNMF expression programs, and at the same time targeted the switched compartment. SERPINE2 was highly expressed in RCC tissues and lowly expressed in para-tumor tissues or HK-2 cell line. SERPINE2 knockdown markedly suppressed RCC cell growth and invasion, while SERPINE2 overexpression dramatically promoted RCC cell metastasis both in vitro and in vivo. In addition, SERPINE2 could activate the epithelial-mesenchymal transition pathway. The above findings demonstrated that the role of distinct expression patterns of malignant cells and TME played a distinct role in RCC progression. SERPINE2 was identified as a potential therapeutic target for inhibiting metastasis in advanced RCC.

## Introduction

Renal cell carcinoma (RCC) is a major malignancy, causing the most common deaths in kidney cancer [1]. Radical surgery is the mainstay of treatment for early RCC at present. However, many RCC patients are already in the advanced stage or accompanied with metastasis at the time of diagnosis, when treatment becomes difficult [2]. Although targeted therapies or immune inhibitors, such as the use of tyrosine kinase inhibitors or immune checkpoint inhibitors treatments have improved the 5-year survival rate in patients with advanced or metastatic RCC, the overall clinical outcomes remain poor [3]. In addition, challenges still exist when clinicians decide on the option of the most effective treatment [4]. Therefore, investigating the underlying mechanisms will be beneficial to identifying potentially effective therapeutic targets for RCC.

Metastasis is the complex progression involving multiple processes and molecular communications between malignant cells and the tumor microenvironment (TME) [5]. Metastasis of RCC and tumor-niche interactions originating from multi-step genetic alternations may lead to abnormal expression of genes such as VHL [6], mTOR [7] and SOX17 [8]. In addition to changes in gene expression at the transcriptional level, chromatin spatial structures [9] and epigenetic factors [10] also contribute to the regulation of genes and their functions via gaining access to spatially distant regulons and regions, thus bringing DNA to sequence-specific binding proteins. However, there is a lack of multi-omics data about RCC metastasis.

In this study, we performed an integrative multi-omics analysis of single-cell RNA-sequencing (scRNA-seq), bulk RNA-sequencing (RNA-seq), 3D high-throughput chromosome conformation capture (Hi-C) and assay for transposable accessible chromatin by high-throughput sequencing (ATAC-seq) for primary and metastatic RCC tumor tissues and compared differences between RCC and normal renal tubular cells. Then, we further validated the level of the identified marker for RCC metastasis by cell functional animal experiments and human tissue microarray. Our data demonstrated that TME played a pivotal role in RCC progression or metastasis and affected the response to immune or targeted therapy in clinical cohorts. More importantly, scRNA-seq identified SERPINE2 as a gene participating in the metastasis process, and hierarchical chromatin organization comparison showed that SERPINE2 was highly-expressed in RCC as a differential expressed gene (DEG) which could potentially predict metastasis, suggesting that it may prove to be a novel target to advanced or metastatic RCC.

## Materials and methods

### Patients and sample collection

**Fig. 1:**
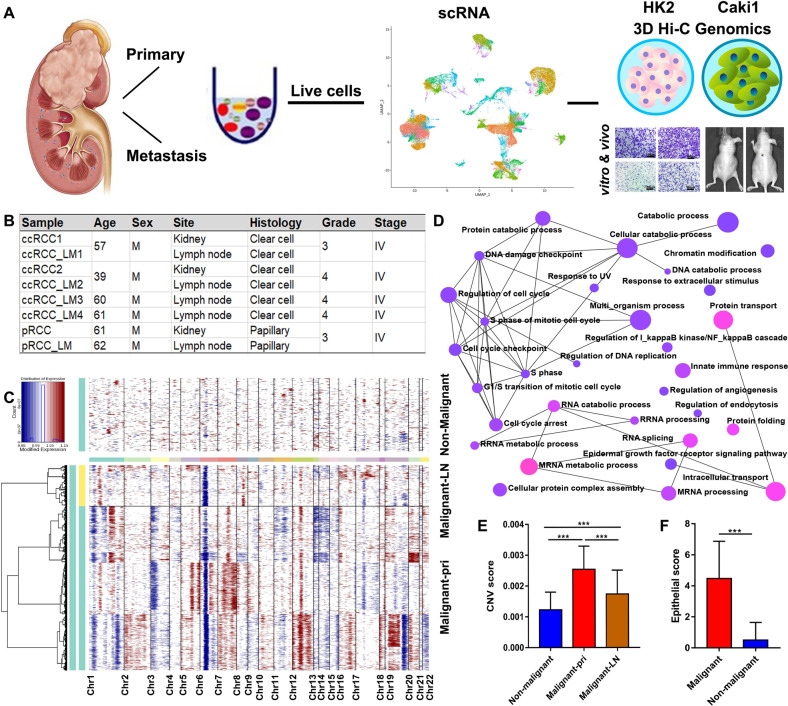
RCC primary and metastatic tumors in scRNA-seq characterization. **A** The workflow of the major genomics approach analysis, involving scRNA-seq and 3D Hi-C technique. **B** The information of patients and tissues for scRNA-seq, including primary tumors from six patients and matched LN metastasis from two of these patients. **C** The heatmap presents the whole CNVs for all individual cells from RCC primary and metastatic tumors, referenced by non-malignant cells. Red: amplifications; Blue: deletions. **D** CNV genes are shown as network with gene-set in GO: BP (https://dev.networkanalyst.ca/NetworkAnalyst). **E** CNV scores of primary (Malignant-pri) and metastatic (Malignant-LN) malignant cells by InferCNV in scRNA-seq analysis. **F** Bar plot presents the epithelial scores (a global epithelial signature) of distinguished as malignant or non-malignant cells according to CNVs. *p < 0.05; **p < 0.01; ***p < 0.001. RCC renal cell carcinoma.

All procedures were approved by the ethical review board of Xinhua Hospital and Third Affiliated Hospital of the Second Military Medical University, and written informed consent was obtained from all included patients. The scRNA-seq samples were obtained from fresh surgically removed tissues of patients with pathologically confirmed diagnosis of advanced RCC, including ccRCC_LM3 and ccRCC_LM4, pRCC_LM used by Bi et al. for RCC study [11]. A total of eight samples from primary and metastatic sites were included for scRNA-seq analysis (Fig. 1B). The two tissue microarrays (TMA-30, *n* = 29 and TMA-2020, *n* = 293) included 322 patients from Xinhua Hospital and Third Affiliated Hospital of the Second Military Medical University (Shanghai, China). The detailed clinical information is presented in Tables 1 and 2. The TCGA sample data were obtained from http://xena.ucsc.edu/ of UCSC Xena, involving bulk-seq, somatic mutation and clinical data.

**Table 1:** Clinical characteristics of patients according to SERPINE2 expression a in Cohort1 (*n* = 29).

Characteristics	SERPINE2 in Cohort1		Sum (*n* = 29)	*p* value
	High expression (*n* = 21)	Low expression (*n* = 8)		
Diagnosis age				0.4270
<60	9	5	14	
≥60	12	3	15	
Gender				0.2832
Male	16	8	24	
Female	5	0	5	
Fuhrman grade				
G1–2	12	6	18	0.6706
G3–4	9	2	11	
TNM stage				0.6472
I–II	16	7	23	
III–IV	5	1	6	
5-year any metastases or recurrence				0.2075
Yes	15	3	18	
No	6	5	11	
5-year overall survival				0.0089
+	17	2	19	
−	4	6	10

**Table 2:** Clinical characteristics of patients according to SERPINE2 expression a in Cohort2 (*n* = 293).

Characteristics	SERPINE2 in Cohort2		Sum (*n* = 293)	*p* value
	High expression (*n* = 112)	Low expression (*n* = 181)		
Diagnosis age				0.8877
<60	69	113	182	
≥60	43	68	111	
Gender				0.3140
Male	77	114	191	
Female	35	67	102	
Fuhrman grade				
G1–2	89	150	239	0.4646
G3–4	23	31	54	
TNM stage				0.0867
I–II	94	164	258	
III–IV	18	17	35	
Any metastases or recurrence				0.0002
Yes	24	12	36	
No	88	169	257	
Overall survival				0.0003
+	97	177	274	
−	15	4	19	

### Sing-cell RNA-sequencing and data processing

Single-cell suspension and droplet-based sequencing were prepared according to the manufacturer’s protocol and our previous work [12]. Seurat (version 3.0.1) was used to perform data quality control [13]. Cells with <200 or >5000 genes or with more than 30% mitochondrial genes were considered low-quality and filtered. Markers of each main cell cluster were identified through FindAllMarker function. Cell type markers were obtained from CellMarker website [14] and previous studies [15, 16]. The characteristic markers used for labeling are presented in Fig. S2.

### InferCNV and cell malignancy evaluation

The InferCNV package was used to investigate the copy number variations (CNVs) by pipeline parameters in scRNA-seq analysis. Each cell was scored based on the range of CNV signals. The CNV signals were defined as previously described [17]. Finally, cells with CNV signal greater than 0.05 and CNV correlation coefficient greater than 0.5 were defined as malignant cells, and cells below these two thresholds were defined as non-malignant cells.

### Epithelial identification and scoring

A series of epithelial markers included EPCAM, cytokeratins and SFN. The mean expression of these genes was used to measure the epithelial score (Table S1).

### Expression program of malignant cell heterogeneity

The non-negative matrix factorization (source: https://github.com/dylkot/cNMF) was applied to classify heterogenic expression programs in malignant cells with the parameters as previously described [17]. Finally, six program clusters were identified and applied to define meta-signatures. The first 20 genes of each cluster were defined as meta-signatures and used to define cell scores. The resulting NNMF program was compared with the meta-program defined in our original analysis, with a global Pearson correlation threshold (across all genes) of 0.2.

### GSEA and GSVA

The samples in TCGA were divided into SERPINE2^high^ and SERPINE2^low^ group. DEGs were calculated by Seurat function. GSEA was used to determine which gene sets were enriched in subgroup comparison. Only a *p* value of a gene less 0.05 was considered as the target of interest. In addition, GSVA was used depending on the C2 and C5 hallmark gene set from molecular signature database, as the instructions of the GSVA package.

### Cell-cell communication analysis

CellphoneDB (based on Python 3.7) [18] and iTALK (R version) [19] are calculation tools for intercellular communication analysis. The primary cell clusters were investigated to establish cell interaction networks. The ligand-receptor pairs with a *p* value < 0.05 were considered to be the interaction of interest.

### Cell line bulk RNA-seq and ATAC-seq

For bulk RNA-seq, total mRNA with poly-A tail was extracted and reverse transcribed to cDNA for sequencing. R software (version 3.6.3) was used for downstream statistical analysis. The Deseq2 (version 1.4.5) R package was used to calculate differential gene expressions. The adjusted *p* value 0.01 and log2 fold change (logFC) >1 were applied to identify significantly DEGs. For ATAC-seq, sequencing was carried out on an Illumina NovaSeq 6000 generating 2 × 75 bp paired-end reads as previously described [20]. ATAC-seq peaks were called using MACS2 with no shifting model, and BEDTools was used to calculate the coverage within peaks.

### 3D high-throughput chromosome conformation capture analysis

All Hi-C sequencing reads were mapped to the human reference genome (hg19) using Bowtie. Raw interaction matrices were normalized by using the iterative correction and eigenvector decomposition (ICE) method and HiCNorm [21]. ICE-normalized interaction matrices at 500-kb resolution were used to detect chromatin compartment types by R-package HiTC. The compartment with a higher gene density was assigned as A compartment (active/euchromatic compartments), and the other compartment was assigned as B compartment (inactive/heterochromatic compartments) [22]. For opologically call associating domains (TADs), ICE-normalized interaction matrices at 40-kb resolution were used by a Perl script matrix2insulation.pl (http://github.com/blajoie/cranenature-2015). A higher resolution was used because TADs are smaller than A/B compartments. Insulation scores (IS) were calculated for each chromosome bin and valleys of IS identified TAD boundaries. TADs smaller than 200 kb or located in telomeres/centromeres were filtered out using the previously described methods. When comparing TADs between two cell lines, at least 70% overlap between two TADs was considered as conserved TADs. Bedtools with the option of “intersectBed -f 0.70-r” was used to identify conserved TADs [23].

### Immune and targeted therapy analysis

Checkmate 025 cohort data [24], including normalized bulk RNA-seq and clinical data, were obtained to perform treatment response analysis in Nivolumab (*n* = 181) and Everolimus (*n* = 130) groups. The TIDE algorithm [25] was used to predict potential ICB response between SERPINE2^high^ and SERPINE2^low^ groups.

### Animals

All animal experiments were approved by the Animal Care and Ethical Committee of the Second Military Medical University (Shanghai, China). The 6-week-old male nude mice were randomly injected with PBS 200 μl containing 1 × 10^6^ 786-O-SERPINE2-OE-PR-luc cells or 786-O-PR-luc via the tail vein. After 8-week injection, the mice were sacrificed and the lung tissue was removed and fixed in 10% formalin buffer solution. 786-O-PR cells were transfected with luciferase reporter gene to detect sub-renal tumor formation or lung metastasis. Tumor growth was monitored weekly using IVIS Lumina imaging system (PerkinElmer, Hopkinton, MA, USA) in vivo bioluminescent optical imaging.

### Cell culture

HK-2, 786-O, 769P, A498, OSRC-2, ACHN, Caki-1 and human umbilical vein endothelial cells were obtained from the Chinese Academy of Sciences (Shanghai, China). Cells were cultured with 1640 or DMEM + 10% fetal bovine serum (FBS) + 1% penicillin at 37 °C and 5% CO_2_.

### Lentiviral gene tool establishment

The overexpression or shRNA lentivirus vector SERPINE2 (OE-SERPINE2 and sh-SERPINE2) was all synthesized by GeneChem Biological Technology (Shanghai, China) using the sequences shown in Table S8. The stable Caki-1 and 786-O cell lines were constructed using lentivirus in which SERPINE2 was knocked down or overexpressed. Lipofectamine 3000 reagent (L3000015, Invitrogen) was used for siRNA and plasmid transfection according to the manufacturer’s protocol.

### Quantitative real-time PCR (qRT-PCR)

Total RNA was extracted using TRIZOL (Invitrogen, USA) and reversed transcribed into cDNA. SYBR Green Real-Time PCR Master Mix (QPK201, Japan) was used to quantify gene transcripts and normalized to the GAPDH expression. The sequences of primers are shown in Table S9.

### Western blot

Total protein was extracted using SDS-PAGE and then transferred to the PVDF membrane (Thermo, USA), which was then incubated with the primary antibodies: SERPINE2 (AB134905, Abcam, USA), GAPDH (#5174, CST, USA), E-cad (#3195, CST, USA), N-cad (#13116, CST, USA), Vimentin (#5741, CST, USA), Snail (#3879, CST, USA), and MMP9 (AB76003, Abcam, USA). The membrane was incubated with a 1:2000 diluted horseradish peroxidase conjugated goat resistant Rabbit (Santa Cruz, USA).

### Cell function assays

For migration and invasion experiments, transwell chambers (Millipore, USA) were used without or with Matrigel (BD Biosciences, USA). Cells were seeded in medium with no FBS into the upper chamber, while the medium with FBS was plated in the lower side. After 3-day seeding, the cells on the lower chamber were fixed with 4% Paraformaldehyde Fix Solution (E672002, Sangon Biotech, Shanghai), stained with crystal violet (E607309, Sangon Biotech), and scanned at ×200 magnification [8]. For clonogenic survival test [23], 100 cells were inoculated in triplicate into each cell of 6-well plates overnight. The transfection portion of the cells was performed as described earlier. After knockdown or overexpression, cells were incubated for 7 days. Subsequently, the colonies were washed with PBS, followed by immobilization with 70% ethanol for 20 min at room temperature and 0.5% crystal violet for 20 min. Colonies with >50 cells were counted under a light microscope. The survival score was calculated as the ratio of plate laying efficiency of treated cells to that of control cells. For CCK8, according to the manufacturer’s instructions, the Cell Counting Kit-8 (CCK8 kit, CK-04, Dojindo, Kumamoto, Kyushu, Japan) was used to detect the proliferation of cells under the conditions shown. Optical density was determined at 450 nm using a microplate reader (EXL800, BioTek Instruments, Winooski, VT, USA).

### Immunohistochemistry (IHC) and H-score

IHC was performed as previously described [26], and the primary antibodies for IHC staining included rabbit anti-SERPINE2 (AB155549, Abcam, USA) and anti-CA9 (AB243660, Abcam, USA). The IHC results were scored as H-score, including semi-quantitative grades by “IHC Profiler (Macro)” of ImageJ (version 1.53a): negative, low-positive, positive, and high-positive. The H-score = 0 * the percentage of negative cells + 1 * the percentage of low positive cells + 2 * the percentage of positive cells + 3 × the percentage of high-positive cells, which ranges from 0 to 300 [27].

### Statistical analysis

Statistical differences between numerical data (mean ± SD) were calculated by Student’s *t* test (two-tailed). Categorical variables were analyzed by chi-square test or Fisher’s exact test. Kaplan–Meier method was used to draw survival curves by “survival” package and “survminer” of R 3.6.3 or GraphPad Prism 7.0 (GraphPad Software, Inc.). ROC analysis was performed to obtain the cut-off value and AUC of H-score. Prognostic accuracy was calculated by Harrell’s concordance index analysis (c-index) with “survcomp” package of R 3.6.3. Nomogram analysis was conducted by “foreign” (version 0.8-78) and “rms” (version 6.0.1) packages for establishing the risk prediction model. All experiments were performed independently at least three times.

## Results

### scRNA-seq landscape of RCC primary and metastatic sites

To investigate the cellular heterogeneity in RCC tumors, the scRNA-seq profiles were presented for primary tumors from six patients and matched LN metastasis from two of these patients (Fig. 1A, B and Tables 1 and 2). Subsequently to quality control, we obtained 46,552 cells from six patients for scRNA-seq landscape. The whole chromosomal inferred copy-number variations (CNVs) across each cell were calculated based on the mean expression among chromosomal regions [17]. The CNVs presented the abnormal profiles among malignant cells, referenced by non-malignant cells (Fig. 1C), which distinguished 30,514 malignant cells and 14,762 non-malignant cells. The CNV genes were shown as the network with gene-set based on GO: BP (Gene Ontology: biological process, Fig. 1D, https://dev.networkanalyst.ca/NetworkAnalyst). The CNV scores of primary (Malignant-pri) and metastatic (Malignant-LN) malignant cells were significantly higher than those of non-malignant cells (*p* < 0.001) (Fig. 1E). Based on the abnormal karyotypes [23], a global expression signature, including EPCAM, cytokeratins and SFN, was established (Table S1). The epithelial score of malignant cells was significantly higher than that of non-malignant cells (*p* < 0.001) (Fig. 1F). According to CNVs and epithelial marker analysis, most cells were part of clusters with a consistent malignant or non-malignant classification.

### RCC TME characterization

**Fig. 2:**
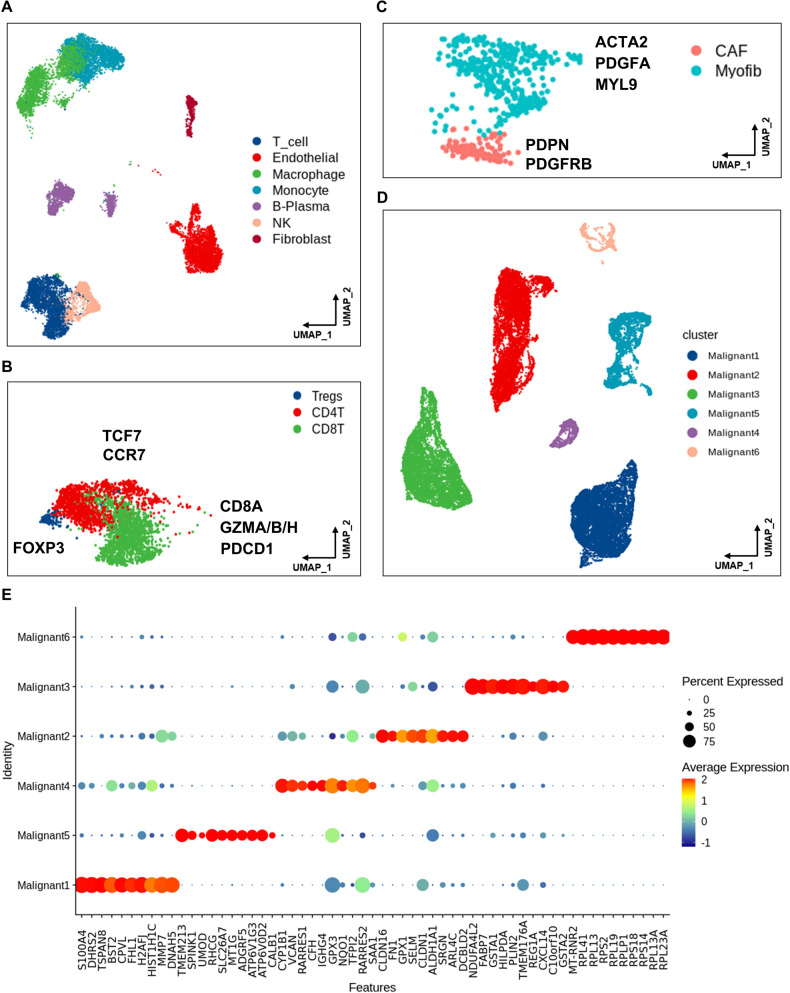
RCC primary and metastatic tumors in scRNA-seq characterization. **A** UMAP plot of non-malignant cells, including T cells, endothelial cells, macrophages, B/Plasma cells, NK cells and fibroblasts identified by maker annotations. **B** Zoomed in UMAP plot of T cell cluster with CD8+ T cells, CD4+ T cells, and Tregs. Marker gens were listed. **C** Zoomed in UMAP plot of Fibroblasts with CAFs and Myofib. Marker genes are listed. **D** UMAP plot of malignant cells shows the Malignant 1–6 subsets. **E** Bubble plot of top 10 genes expression in Malignant 1–6 subsets; the size of bubble represents the percent expressed of cells; the color represents the mean expression level of each gene in clusters: red means the high expression.

The 14,762 non-malignant cells were clustered into seven main cell types (Fig. 2A), involving T cells, endothelial cells, macrophages, B/Plasma cells, NK cells and fibroblasts identified by maker annotations (Table S2). The T cells and fibroblasts subgroups were further clustered at a higher resolution. As shown in Fig. 2B, the major T cell cluster was grouped into CD8+ T cells (expressed both cytotoxic and exhausted marker), CD4+ T cells (classical marker, TCF7 and CCR7), and regulatory T cells (Tregs, FOXP3). The 540 fibroblasts were re-clustered into two subgroups (Fig. 2C). The one expressed conventional myofibroblast markers ACTA2 and MYL9. Myofibroblasts are acknowledged as components of TME and can be detected in both primary and metastatic niche [28]. The other subset presented the characterization of cancer-associated fibroblasts (CAFs), expressing markers such as PDPN and PDGFRB [29]. Meanwhile, these subsets expressed markers with immediate early response genes (e.g., JUN, FOS), mesenchymal markers (e.g., VIM, THY1) and ECM (MMP11) (Fig. S1A). This heterogeneity of fibroblasts among tumors was consistent with the perspective that CAF is involved in complex structural and paracrine interactions in TME. The cell-cell communication result also showed frequent interactions between CAF and other cells (Fig. S1B, C). Compared with non-malignant cells, 30,514 malignant cells were clustered based on the tumor heterogeneity in monocle UMAP (Fig. 2D). The top 10 expressed markers of each malignant cell cluster are shown in Fig. 2E.

### Heterogeneity of the malignant cell expression patterns and identification of the metastasis programs

**Fig. 3:**
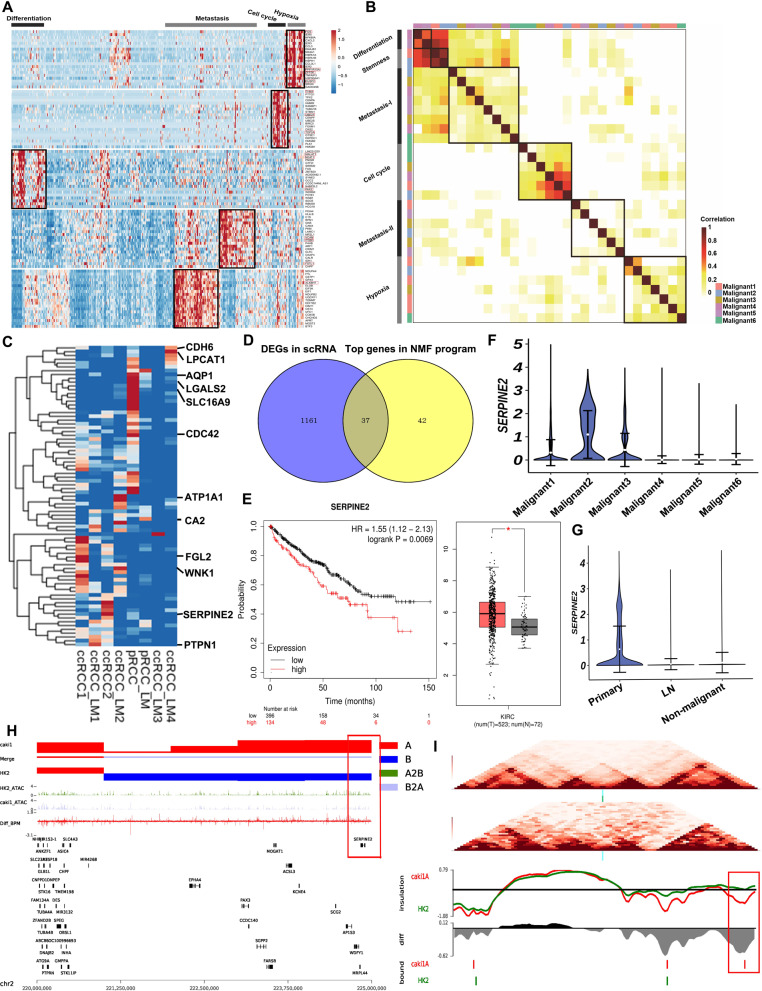
NNMF clustering characterizing heterogenic malignant cells and identifying metastasis program in RCC tumors. **A** The heatmap presents DEGs (rows) identified by NMF, which are clustered by expression in each cell (column) of the representative Malignant 4. The gene signature was identified and indicated on the top. **B** The heatmap shows the correlations of 60 intratumoral procedures from six malignant subsets. The clustering identified six coherent expression programs across tumors. **C** The heatmap depicts genes scores by each sample (column) for genes (row) in the Metastasis-II program. **D** Venn plot shows the number of DEGs (malignant vs. non-malignant cells in scRNA profile) and the top genes in the NNMF program. **E** The KM survival curve of SERPINE2-high and SERPINE2-low group in TCGC KIRC (Left, *n* = 530); the SERPINE2 expression between tumor tissues (*n* = 530) and normal tissues (*n* = 72) in KIRC (Right). *p < 0.05; **p < 0.01; ***p < 0.001. **F** Violin plot shows the SERPINE2 expression among Malignant 1–6 subsets. **G** Violin plot shows the SERPINE2 expression among cells from primary and metastatic tumors and non-malignant subsets. **H** The comprehensive plot of Caki-1 and HK-2 cells shows the compartment status at the SERPINE2 locus B to A, and this locus at ATAC-seq presents an open status. The red bar represents A compartment; Blue bar represents B compartment. Merge bar represents the compartment status alternations. The Diff_BPM shows all the DEGs between the two cell lines in this chromatin region. **I** The TAD analysis shows TAD boundaries reforming/disappearing and insulation scores between HK-2 and Caki-1. Red line, for Caki-1; Green line, for HK2. Caki-1 shows lower insulation score than HK-2 do, and new boundary reforms compared with HK-2. DEGs differentially expressed genes.

Next, we investigated how the expression patterns varied in heterogenetic malignant cells among the tumor subsets by focusing on eight tumors that acquired the most of malignant cells. The NNMF algorithm was used to reveal consistent sets of genes tendentiously co-expressed by clusters of malignant cells. We identified four gene sets that varies among cells of Malignant 4 cluster cells (Figs. 3A and S2A). Using this method to each of the six malignant clusters, we identified a total of 60 gene signatures that varied consistently among individual cells in at least one malignant cluster (Table S3). Then, we applied hierarchical clustering to extract the 60 gene signatures into meta-signatures that represented different co-expression patterns in multiple malignant subsets (Fig. 3B). The high degree of consistency among signatures of different malignant cluster cells suggested that they embraced a common state of expression heterogeneity within tumors.

Six expression patterns were tendentiously expressed by at least two subsets of malignant cells. As shown in Fig. 3B, two programs (clusters 1 and 2) were mainly composed of epithelial genes such as cytokeratin and EPCAM (Table S4). Although epithelial markers were expressed in all malignant cells, many of them were generally consistent in malignant cells (Table S4), and could reflect the level of epithelial differentiation and stemness. One additional program reflected G1/S and G2/M phases of the cell cycle and distinguished different cycle cells in each malignant subset. The last procedure enriched hypoxic-related genes (Fig. 3B).

We focused on the expression program related to metastasis in clusters 3 and 5 (Fig. 3B). The metastasis-I program contained ECM-related genes and had characteristics of EMT (Table S5). This process was evident in four subsets across all the six malignant subsets examined (Fig. S2B). Although EMT has been widely recognized as the potential target of drug resistance and tumor cell invasion and metastasis, their pattern and significance in human epithelial tumors are ambiguous. We then further examined the Metastasis-II program for EMT pattern. Besides ECM genes, this program included the partial EMT hallmarks such as conventional ITGA3, but lacked other markers (SNAIL, ZO1). The overall expression of epithelial markers remained remarkably unchanged, while the signatures were accompanied by decreased expression of some epithelial genes (Fig. S2C), suggesting that the Metastasis-II may be an unclassical EMT program.

### SERPINE2 plays a key role in the metastasis program

We then examined the expression of genes involved in the Metastasis-II program across all the RCC samples (Table S6). These annotated genes were expressed in all RCC tissues, including primary and metastatic samples, and were the top genes in at least two samples (Fig. 3C). We then detected DEGs between malignant and non-malignant cells in the scRNA profile (Table S7) and found 37 commonly expressed genes, the top genes of which were in the NNMF program (Fig. 3D). Then, we examined all common genes in the TCGA survival data to filter out the oncogenes (Figs. 3E and S3A), knowing that oncogenes are relevant to poor survival outcomes [30] and highly expressed in tumor samples [31] compared with the normal tissues (Fig. S3B). After filtration, the highly-expressed SERPINE2 was found to be associated with the worse overall survival (OS) (Fig. 3E, *p* = 0.0069, HR = 1.55 (1.12–2.13) and *p* < 0.05). All the malignant scRNA subsets expressed SERPINE2, and the Malignant 1–3 had even higher expression (Fig. 3F). In addition, primary malignant cells expressed the highest SERPINE2 (Fig. 3G). Both GSEA and GSAV indicated that cell proliferation, cell movement and migration pathways were significantly enriched in SERPINE2-high malignant cells (Fig. S4A, B). The malignant cells frequently contacted with other cell clusters (Fig. S1B), and the growth factor-related ligands and receptors were actively expressed in malignant cells (Fig. S1C).

Next, we compared the SERPINE2 expression in RCC cell lines and the normal renal epithelial cell line HK-2. It was found that Caki-1 expressed the highest SERPINE2, compared with the other RCC cell lines (Fig. S4C). Knowing that Caki-1 originates from the human RCC metastatic tumor site, we performed Hi-C, ATAC-seq, combined with RNA-seq between Caki-1 and HK-2 to explore whether the chromatin spatial structures and epigenetic factors contributed to gene and function regulation in SERPINE2. The compartment status at the SERPINE2 locus was B (normal cell line) to A (RCC cell line), and this locus at ATAC-seq presented open status, suggesting that the SERPINE2 locus presented the active interaction frequency (Fig. 3H). The RNA-seq result also showed that SERPINE2 was highly expressed in Caki-1 compared with that in HK-2. Moreover, the region of SERPINE2 presented the TAD differences between HK-2 and Caki-1, suggesting that the TAD size decreased with the generation of new TAD boundaries in Caki-1 cells (Fig. 3I). These data show that SERPINE2 not only worked at the transcriptomic level but was correlated with the genomics alternations to RCC. Thus, SERPINE2 may play a potential role in RCC metastasis based on the whole-genomics and scRNA-seq.

### SERPINE2 serves as a metastasis-associated oncogene in RCC and drug response

**Fig. 4:**
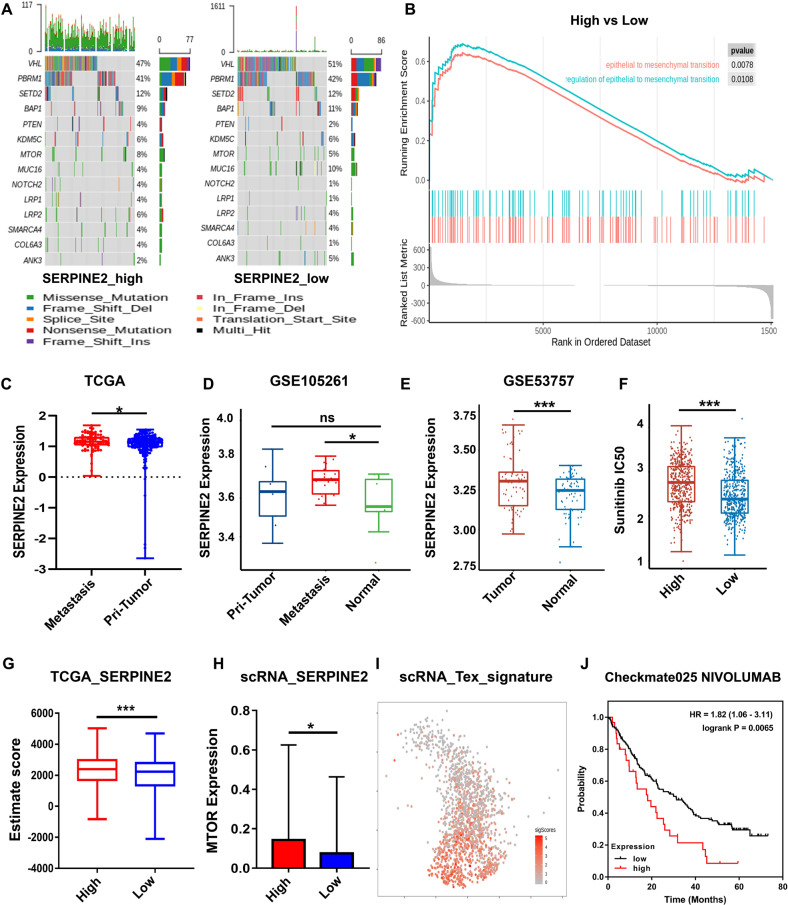
SERPINE2 serves as a metastasis-associated oncogene in RCC and drug response. **A** The Oncoprint of conventional marker genes of RCC with alterations in SERPINE2 high and low groups. Tumor mutation burden is represented for individual samples as a bar chart above the oncoprint. **B** The GSEA plot shows enrichment of EMT-related pathways in SERPINE2 high group in TCGA. FDR < 0.05 is considered as significantly enriched. **C** SERPINE2 expression between tumor tissues from patients with metastasis (positive N stage or M stage >0, *n* = 89) and from patients with no metastasis (N stage or M stage = 0, *n* = 199). Data seen in Table S10. **D** SERPINE2 expression among primary (*n* = 9), metastatic tumors (*n* = 26) and normal tissues (*n* = 9) in GSE105261. Data were obtained from https://www.aclbi.com/static/index.html#/geo. **E** SERPINE2 expression between tumors (*n* = 72) and normal tissues (*n* = 72) in GSE53757. Data were obtained from https://www.aclbi.com/static/index.html#/geo. **F** IC50 prediction of sunitinib for SERPINE2 high and low groups in TCGA, based on the GDSC database. **G** The estimate score of tumor tissues from SERPINE2 high and low groups, reflecting the immune infiltration. **H** MTOR expression from cells in scRNA-seq analysis (50%: 50%). **I** UMAP plot shows the terminally exhausted CD8+ T cell signature expression. **J** The KM survival curve of SERPINE2-high and SERPINE2-low groups in the cohort of clinical trials Checkmate025 with Nivolumab. *p < 0.05; **p < 0.01; ***p < 0.001.

SERPINE2 is a type of secreted protein and an inhibitor of plasminogen, urokinase and thrombin [32]. Previous studies have shown that SERPINE2 expression is associated with tumorigenesis and tumor cell invasion [33, 34]. SERPINE2 is also commonly upregulated in lung [35], colorectal [36] and pancreatic [37] carcinoma. However, the molecular mechanisms by which SERPINE2 enhances tumor metastasis remains unclear. Firstly, we assessed somatic alterations in TCGA patients based on the mean expression of SERPINE2 (high group, *n* = 164; low group, *n* = 168). Genetic changes of previous studies on RCC are shown in Fig. 4A. The MTOR, MUC16, NOTCH2 and LPR1 presented differences in SERPINE2 high and low groups (Fig. 4A). Then, GSEA was performed between the two groups in TCGA. The EMT pathways were significantly enriched with *p* value <0.05 of false discovery rate (Fig. 4B). SERPINE2 was highly expressed in the tissues from patients with LN or other metastases (*n* = 89) as compared with that in the primary tumor tissues with no metastasis (*n* = 199) (Fig. 4C, *p* < 0.05). In addition, SERPINE2 of expression in patients with metastasis was also significantly higher than that in the normal tissues of the GSE105261 cohort (*p* < 0.05) (Fig. 4D). SERPINE2 expression in tumors was significantly higher than that in normal tissues in the GSE53757 cohort (*p* < 0.001) (Fig. 4E). The similar result was also observed in the GSE40435 and GSE22541 cohorts (*p* < 0.001, *p* < 0.01) (Fig. S4A). Next, we predicted the drug response for TCGA samples based on the largest publicly available pharmacogenomics database, the Genomics of Drug Sensitivity in Cancer (cgp2016), https://www.cancerrxgene.org/. The SERPINE2-high group had higher IC50 in response to sunitinib, which means a lower response rate compared with the SERPINE2-low group (*p* < 0.001) (Fig. 4F). Estimate algorithm could calculate the immune infiltration level of bulk-seq sample. We found that the SERPINE2-high group had higher immune infiltration than SERPINE2-low group (*p* < 0.001) (Fig. 4G). CD8+ T cell expression was negatively correlated with SERPINE2 expression in EIPC (*p* < 0.05) (Fig. S5B), or CIBERSORTx (*p* < 0.01) (Fig. S5C) calculation. Thus, SERPINE2 expression was likely to be associated with immunotherapy, too. Then, we detected the expression of MTOR expression in scRNA-seq. The SERPINE2-high group expressed more MTOR (*p* < 0.05) (Fig. 4H). However, the SERPINE2 level was not related to the survival in the Checkmate025 cohort treated with everolimus (*p* = 0.0629, HR = 0.68, 0.43–1.07) (Fig. S5D). The signature (PDCD1, HAVCR2, LAG3, TIGIT, TOX, ENTPD1, BATF and PRDM1) of terminally exhausted CD8+ T cells integrated in Fig. 4I suggested that the terminally exhausted CD8+ T cells actually affected immune infiltration. The higher SERPINE2 expression was associated with the worse survival in the Checkmate025 cohort treated with nivolumab (*p* = 0.0065, HR = 1.82, 1.06–3.11) (Fig. 4J). These data suggest that SERPINE2 may be a malignant gene in RCC and participate in drug response.

### SERPINE2 promotes ccRCC invasion in vitro and vivo, accompanied by epithelial-mesenchymal transition (EMT)

**Fig. 5:**
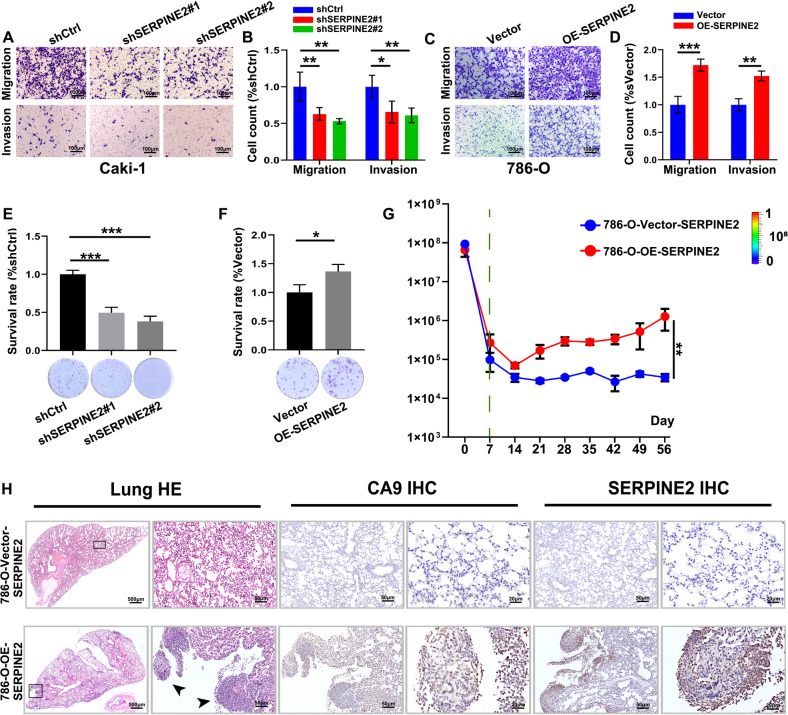
SERPINE2 promotes ccRCC progression, accompanied by EMT. **A**, **B** Representative images of transwell assay without or with Matrigel shows the migration and invasion abilities among shCtrl, shSERPINE2#1 and shSERPINE2#2 group in Caki-1 cells, relative change compared with shCtrl group. Scale bar = 100 μm. **C**, **D** Representative images of transwell assay without or with Matrigel shows the migration and invasion abilities between Vector, OE-SERPINE2 group in 786-O cell, relative change compared with Vector group. Scale bar = 100 μm. **E** The cell survival rate among shCtrl, shSERPINE2#1 and shSERPINE2#2 group in Caki-1 cells, relative change compared with shCtrl group. **F** The cell survival rate among Vector, OE-SERPINE2 group in 786-O cell, relative change compared with Vector group. **G** Whole body bioluminescence (photons/second) following tail vein injection of cells in mice. Differences occurred from day 7 time point (Green line). **H** The lung tissue of 786-O-OE-SERPINE2 or 786-O-NC-SERPINE2 of mice; the representative lung metastasis site by HE staining. Scale bar = 500 μm; Scale bar = 50 μm. The IHC results of CA9 and SERPINE2; Scale bar = 50 μm; Scale bar = 20 μm. EMT epithelial-mesenchymal transition.

To investigate whether SERPINE2 promoted the tumor malignant biological behavior and metastasis in RCC, we validated the function of SERPINE2 in RCC by qRT-PCR. The results showed that the expression of SERPINE2 was high in Caki-1 and low in 786-O cells (Fig. S4C). Then, SERPINE2 overexpression cells (786-O) and SERPINE2 knockdown cells (Caki-1) were constructed and observed by qPCR and Western blot (Figs. 5G and S6A–C). The effect of SERPINE2 expression on RCC cell proliferation and invasion was examined via colony formation and CCK-8 cell proliferation assay, Transwell cell migration and invasion assay. The results showed that RCC cell migration and invasion were significantly decreased in sh-SERPINE2 group (Fig. 5A, B, *p* < 0.01, *p* < 0.01; Fig. 5A, B, *p* < 0.01, *p* < 0.05), and significantly increased in OE-SERPINE2 group (Fig. 5C, D, *p* < 0.001; Fig. 5C, D, *p* < 0.01) in 48 h. The cell survival rate was significantly decreased in sh-SERPINE2 group (*p* < 0.01) (Fig. 5E), and significantly increased in OE-SERPINE2 group (*p* < 0.05) (Fig. 5F). Knowing that change in ECM expression and matrix metalloproteinase (MMP) participates in cancer cell metastasis and SERPINE2 enhances pancreatic tumor invasion [37] and lung metastasis of breast cancer via producing ECM and secreting MMP-9 [38], we next detected the expression of EMT markers (E-cadrin, N-cadrin, VIM and Snail) and MMP-9. qPCR and Western blot results showed that the EMT activation markers were highly-expressed in OE-SERPINE2 cells (*p* < 0.01) (Fig. S6A–C, Supplementary Materials), and significantly decreased in sh-SERPINE2 cells (*p* < 0.01) (Fig. S6A–C). Moreover, 786-O-OE-SERPINE2 or 786-O-NC-SERPINE2 cells labeled with stable luciferase were injected into the caudal veins of nude mice. The photon flux was recorded during the 8 weeks. It was found that lung metastasis was more serious in mice of 786-O-OE-SERPINE2 group as compared with the mice in the control group (*p* < 0.01) (Figs. 5G and S6F). The HE results of lung tissues are presented in Fig. 5H. The Carbonic Anhydrase 9 (CA9) and SERPINE2 were highly expressed in the 786-O-OE-SERPINE2 induced lung metastasis sites (Fig. 5H).

### SERPINE2 is associated with poor survival and predicts RCC metastasis in our clinical cohort

**Fig. 6:**
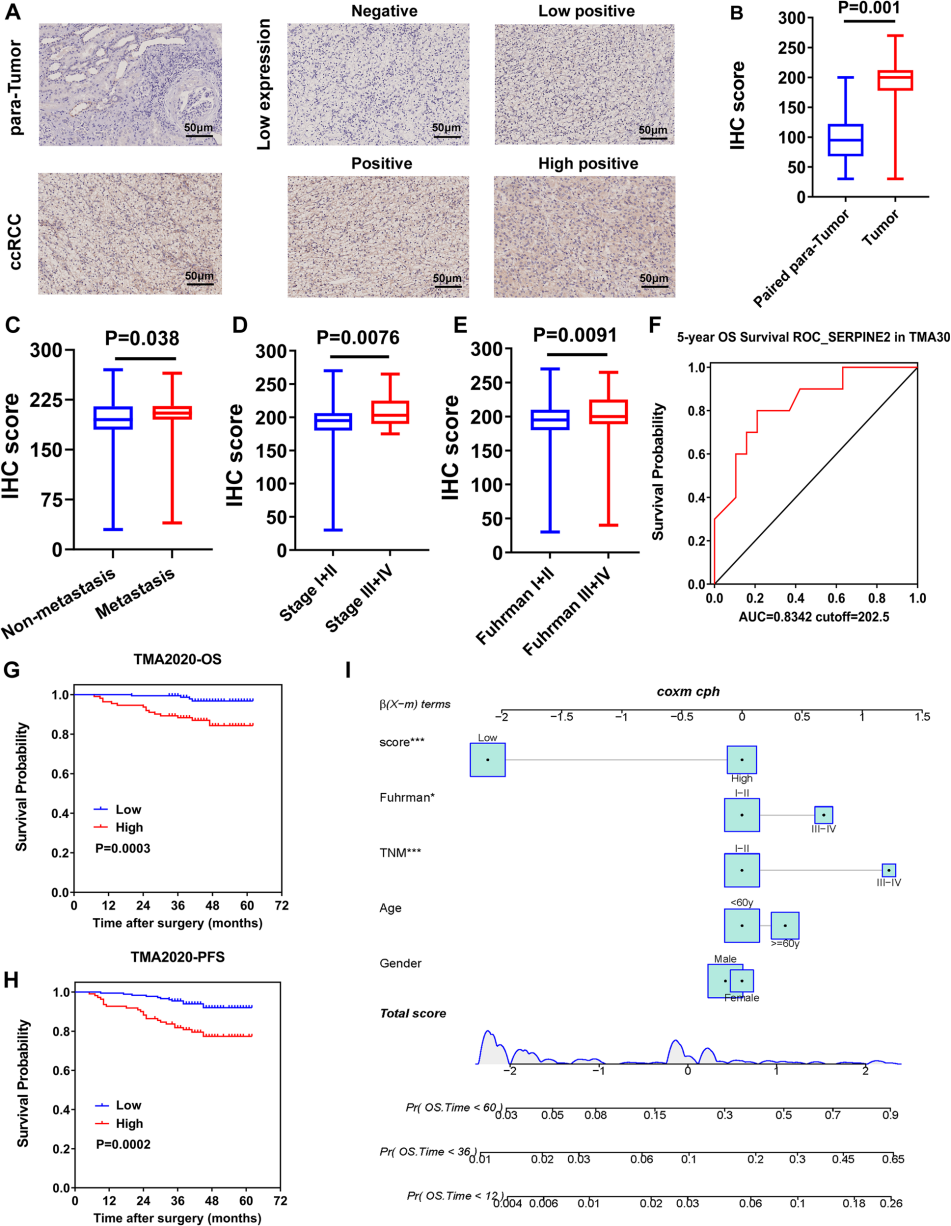
SERPINE2 predicts poor survival and RCC metastasis in our clinical cohort. **A** Representative IHC staining and H-score for SERPINE2 in RCC tissues and matched para-tumor tissues (scale bar = 50 μm). **B** IHC score between tumor tissues and matched para-tumor tissues. **C** IHC score between tumor tissues from patients with metastasis and no metastasis. **D** IHC score between patients with stage I, II and stage III, IV. **E** IHC score between patients with Fuhrman I, II and Fuhrman III, IV. **F** The ROC curve for SERPINE2 expression in 5-year OS (TMA30 cohort). **G** The KM curve for OS difference between SERPINE2 high and low groups. **H** The KM curve for PFS difference between SERPINE2 high and low groups. **I** The nomogram for 1-, 3- and 5-year OS. OS overall survival, PFS progression-free survival.

We validated the expression in tissue microarrays of two cohorts (TMA30, *n* = 29; TMA2020, *n* = 289) by IHC. H-score quantitative analysis (Fig. 6A) showed that the expression of SERPINE2 in tumor tissue group was higher than that in para-tumor tissue group (*p* = 0.001) (Fig. 6B). We classified the cohorts according to the clinical information and found that SERPINE2 expression was significantly higher in the tissues with metastasis (*p* = 0.038) (Fig. 6C) and a higher Fuhrman grade (*p* = 0.0091) (Fig. 6D) or tumor stage (*p* = 0.0076) (Fig. 6E) based on the IHC score. According to the optimal cut-off values from ROC analysis in SERPINE2 using 5-year OS of TMA30, the patients were classified as a high-expression group and a low-expression group. The cut-off value of H-score was 202.5 with AUC of 0.8342 (Fig. 6F). We wondered whether the expression levels of SERPINE2 reflected the corresponding clinical outcomes. The result of Kaplan–Meier survival analysis showed that patients in SERPINE2 -high subgroup had poorer OS (Fig. 6G, *p* = 0.0003) and progress-free survival (PFS) (*p* = 0.0002) (Fig. 6H). Then, we performed the nomogram analysis for the clinical information and SERPINE2 (Figs. 6I and S7A, B) and found that SERPINE2 expression was an independent risk factor of OS of ccRCC patients.

## Discussion

The therapeutic challenges of advanced or metastatic RCC have urged researchers to explore the underlying mechanisms leading to RCC progression. The heterogeneity among solid malignancies drives one of the major challenges. scRNA-seq can help identify TME distinction [39], developmental patterns [40, 41], drug response/resistance characterization [42], and immune infiltration programs involved with tumor behaviors and clinical treatment [43, 44]. On the other hand, although some somatic analyses have been performed for RCC, tumorigenesis related DNA accessibility and gene localization remains unclear. In this study, we identified primary RCC tumors and matched LN metastases by using this well-integrated multi-omics analysis. Our study revealed a metastasis program and SERPINE2 associated with this pattern across multiple tumors, involving significant structural variation of RCC cell line and obvious correlation between RCC and TME.

EMT is a classical and acknowledged driver of metastasis, which is regulated by different activators at different levels [45]. In this study, we identified a Metastasis-II program in malignant cells, an unconventional pattern, compared with Metastasis-I program (with classical EMT modules). Although Metastasis-II program involves certain EMT-like enriched alternations of epithelial and mesenchymal genes, it lacks the expression of classical TFs of EMT such as SNAIL, SLUG and TWIST [46] in scRNA-seq analysis and the vitro experiment (Figs. 5G and S2F). Similarly, SERPINE2, as a key oncogene in this program validated by multi-omics, whose molecules expressions brought this situation in vitro. SERPINE2 activated the process by decreasing the expression of the epithelial marker E-cadherin and increasing the level of the interstitial markers N-cadherin and Vimentin, which further stimulated MMP9 expression to invade ECM. This is consistent with the finding in other types of malignancies [38, 47].

Considering the lack of conventional regulation patterns, the Metastasis-II pattern reflects an additional state that generalizes some aspects of EMT. Actually, the expression characterization of EMT has aroused increasing attention because growing evidence has demonstrated the role EMT at different stages [48]. It is also speculated that the dynamic EMT stages embrace both invasiveness and tumor development [49]. Moreover, it is still ambiguous whether a complete EMT process undergoes or whether the several metastasis programs coexist in RCC metastasis. In any case, our identification of SERPINE2 from an unbiased analysis for malignant cells of RCC patients provides a novel sight into the programs of human cancers and metastasis for future research.

ECM remodeling usually requires proteases and their inhibitors to induce tumor metastasis. It was found in our study that SERPINE2 expression was upregulated in RCC. It is for the first time that we unveiled the role of SERPINE2 in RCC, demonstrating that increased expression of SERPINE2 significantly decreased OS and PFS. SERPINE2 overexpression promoted RCC cell migration and invasion, and enhanced lung metastasis in mice in vivo, while SERPINE2 did not affect cell proliferation in the 48-h CCK assay (Fig. S6D, E). This potentially suggests that SERPINE2 may be able to promote invasion rather cell growth. SERPINE2 has been reported to participate in metastasis of several human cancers through various mechanisms, such as by re-establishing tumor stroma and the polarization of TAM [50], or activation of glycogen synthesis kinase 3β [47] and P38 [51] pathways. Various cytokines such as fibroblast growth factor (FGF) [23] and transforming growth factor β (TGFβ) [52] are known to stimulate the secretion of SERPINE2 in normal cell lines. Here, we also found that FGF, VEGF, TGFβ and their receptors were activated in cell-cell communication (Fig. S1B), suggesting that SERPINE2 may be involved in promoting cytokines-related metastasis by cell-cell interaction. Bulk-seq analysis has been performed for several tumor types to classify subtypes but failed to describe the heterogeneity within tumors. It was found in our study that the mesenchymal subtypes could reflect TME. The potential of stromal components suggests that future subtype systems may eventually need to integrate malignant and non-malignant components together.

In conclusion, our results described a landscape of malignant, stromal and immune cells for RCC and matched LN. Our identification of the Metastasis-II program and SERPINE2 by multi-omics may facilitate linking the unclassical EMT data to the RCC biology in vivo and vitro. Although further research is needed, the relation of this metastasis program and SERPINE2 level to worse clinical features may help develop new diagnostic and treatment strategies for advanced RCC.

## Supplementary information

Supplementary figures

Supplementary figure legends

Table. S1

Table. S2

Table. S3

Table. S4

Table. S5

Table. S6

Table. S7

Table. S8

Table. S9

Table. S10

Extended Data

aj-checklist

## Data Availability

All primary data presented in this study are available from the corresponding author upon reasonable request and we would upload the scRNA-seq, Hi-C, ATAC-seq and RNA-seq raw data to the Genome Sequence Archive of Human in the BIG Data Center, Chinese Academy of Sciences under accession codes HRA000454 for that are publicly accessible at https://bigd.big.ac.cn/gsa-human. Processed data could be accessed at 10.6084/m9.figshare.21625964 after reasonable request.

## Code availability

Codes could be accessed along with the supporting data upon reasonable request from Wen-jC or X-gC.

## References

[1] Barata PC, Rini BI. Treatment of renal cell carcinoma: current status and future directions. CA Cancer J Clin. 2017;67:507–24. 28961310 10.3322/caac.21411

[2] Dabestani S, Marconi L, Hofmann F, Stewart F, Lam TBL, Canfield SE, et al. Local treatments for metastases of renal cell carcinoma: a systematic review. Lancet Oncol. 2014;15:e549–61. 25439697 10.1016/S1470-2045(14)70235-9

[3] Hsieh JJ, Purdue MP, Signoretti S, Swanton C, Albiges L, Schmidinger M, et al. Renal cell carcinoma. Nat Rev Dis Prim. 2017;3:17009. 28276433 10.1038/nrdp.2017.9 PMC5936048

[4] Riaz IB, He H, Ryu AJ, Siddiqi R, Naqvi SAA, Yao Y, et al. A living, interactive systematic review and network meta-analysis of first-line treatment of metastatic renal cell carcinoma. Eur Urol. 2021;80:712–23. 33824031 10.1016/j.eururo.2021.03.016

[5] Satcher RL, Zhang XHF. Evolving cancer-niche interactions and therapeutic targets during bone metastasis. Nat Rev Cancer. 2022;22:85–101. 10.1038/s41568-021-00406-5. 34611349 10.1038/s41568-021-00406-5 PMC10281546

[6] Shenoy N, Pagliaro L. Sequential pathogenesis of metastatic VHL mutant clear cell renal cell carcinoma: putting it together with a translational perspective. Ann Oncol. 2016;27:1685–95. 27329246 10.1093/annonc/mdw241

[7] Kim K, Zhou Q, Christie A, Stevens C, Ma Y, Onabolu O, et al. Determinants of renal cell carcinoma invasion and metastatic competence. Nat Commun. 2021;12:5760. 34608135 10.1038/s41467-021-25918-4 PMC8490399

[8] Wang C, Wang Y, Hong T, Ye J, Chu C, Zuo L, et al. Targeting a positive regulatory loop in the tumor-macrophage interaction impairs the progression of clear cell renal cell carcinoma. Cell Death Differ. 2021;28:932–51. 33009518 10.1038/s41418-020-00626-6 PMC7937678

[9] Vian L, Pękowska A, Rao SSP, Kieffer-Kwon K-R, Jung S, Baranello L, et al. The energetics and physiological impact of cohesin extrusion. Cell. 2018;173:1165–78.e20. 29706548 10.1016/j.cell.2018.03.072 PMC6065110

[10] Buenrostro JD, Giresi PG, Zaba LC, Chang HY, Greenleaf WJ. Transposition of native chromatin for fast and sensitive epigenomic profiling of open chromatin, DNA-binding proteins and nucleosome position. Nat Methods. 2013;10:1213–8. 24097267 10.1038/nmeth.2688 PMC3959825

[11] Bi K, He MX, Bakouny Z, Kanodia A, Napolitano S, Wu J, et al. Tumor and immune reprogramming during immunotherapy in advanced renal cell carcinoma. Cancer Cell. 2021;39:649–61.e5. 33711272 10.1016/j.ccell.2021.02.015 PMC8115394

[12] Pan X-W, Zhang H, Xu D, Chen J-X, Chen W-J, Gan S-S, et al. Identification of a novel cancer stem cell subpopulation that promotes progression of human fatal renal cell carcinoma by single-cell RNA-seq analysis. Int J Biol Sci. 2020;16:3149–62. 33162821 10.7150/ijbs.46645 PMC7645996

[13] Butler A, Hoffman P, Smibert P, Papalexi E, Satija R. Integrating single-cell transcriptomic data across different conditions, technologies, and species. Nat Biotechnol. 2018;36:411–20. 29608179 10.1038/nbt.4096 PMC6700744

[14] Zhang X, Lan Y, Xu J, Quan F, Zhao E, Deng C, et al. CellMarker: a manually curated resource of cell markers in human and mouse. Nucleic Acids Res. 2019;47:D721–8. 30289549 10.1093/nar/gky900 PMC6323899

[15] Hu J, Chen Z, Bao L, Zhou L, Hou Y, Liu L, et al. Single-cell transcriptome analysis reveals intratumoral heterogeneity in ccRCC, which results in different clinical outcomes. Mol Ther. 2020;28:1658–72. 32396851 10.1016/j.ymthe.2020.04.023 PMC7335756

[16] Young MD, Mitchell TJ, Vieira Braga FA, Tran MGB, Stewart BJ, Ferdinand JR, et al. Single-cell transcriptomes from human kidneys reveal the cellular identity of renal tumors. Science. 2018;361:594–9. 30093597 10.1126/science.aat1699 PMC6104812

[17] Puram SV, Tirosh I, Parikh AS, Patel AP, Yizhak K, Gillespie S, et al. Single-cell transcriptomic analysis of primary and metastatic tumor ecosystems in head and neck cancer. Cell. 2017;171:1611–24.e24. 29198524 10.1016/j.cell.2017.10.044 PMC5878932

[18] Vento-Tormo R, Efremova M, Botting RA, Turco MY, Vento-Tormo M, Meyer KB, et al. Single-cell reconstruction of the early maternal-fetal interface in humans. Nature. 2018;563:347–53. 30429548 10.1038/s41586-018-0698-6 PMC7612850

[19] Wang Y, Wang R, Zhang S, Song S, Jiang C, Han G, et al. iTALK: an R package to characterize and illustrate intercellular communication. bioRxiv. 2019:507871.

[20] Mezger A, Klemm S, Mann I, Brower K, Mir A, Bostick M, et al. High-throughput chromatin accessibility profiling at single-cell resolution. Nat Commun. 2018;9:3647. 30194434 10.1038/s41467-018-05887-x PMC6128862

[21] Imakaev M, Fudenberg G, McCord RP, Naumova N, Goloborodko A, Lajoie BR, et al. Iterative correction of Hi-C data reveals hallmarks of chromosome organization. Nat Methods. 2012;9:999–1003. 22941365 10.1038/nmeth.2148 PMC3816492

[22] Gibcus JH, Samejima K, Goloborodko A, Samejima I, Naumova N, Nuebler J, et al. A pathway for mitotic chromosome formation. Science. 2018;359:6376. 10.1126/science.aao6135 PMC5924687 29348367

[23] Acosta H, Iliev D, Grahn THM, Gouignard N, Maccarana M, Griesbach J, et al. The serpin PN1 is a feedback regulator of FGF signaling in germ layer and primary axis formation. Development. 2015;142:1146–58. 25758225 10.1242/dev.113886

[24] Braun DA, Street K, Burke KP, Cookmeyer DL, Denize T, Pedersen CB, et al. Progressive immune dysfunction with advancing disease stage in renal cell carcinoma. Cancer Cell. 2021;39:632–48.e8. 33711273 10.1016/j.ccell.2021.02.013 PMC8138872

[25] Jiang P, Gu S, Pan D, Fu J, Sahu A, Hu X, et al. Signatures of T cell dysfunction and exclusion predict cancer immunotherapy response. Nat Med. 2018;24:1550–8. 30127393 10.1038/s41591-018-0136-1 PMC6487502

[26] Wang C, Li Y, Chu C-M, Zhang X-M, Ma J, Huang H, et al. Gankyrin is a novel biomarker for disease progression and prognosis of patients with renal cell carcinoma. EBioMedicine. 2019;39:255–64. 30558998 10.1016/j.ebiom.2018.12.011 PMC6354735

[27] Viola P, Maurya M, Croud J, Gazdova J, Suleman N, Lim E, et al. A validation study for the use of ROS1 immunohistochemical staining in screening for ROS1 translocations in lung cancer. J Thorac Oncol. 2016;11:1029–39. 27179848 10.1016/j.jtho.2016.03.019

[28] Pelon F, Bourachot B, Kieffer Y, Magagna I, Mermet-Meillon F, Bonnet I, et al. Cancer-associated fibroblast heterogeneity in axillary lymph nodes drives metastases in breast cancer through complementary mechanisms. Nat Commun. 2020;11:404. 31964880 10.1038/s41467-019-14134-w PMC6972713

[29] Madar S, Goldstein I, Rotter V. ‘Cancer associated fibroblasts’-more than meets the eye. Trends Mol Med. 2013;19:447–53. 23769623 10.1016/j.molmed.2013.05.004

[30] Nagy Á, Munkácsy G, Győrffy B. Pancancer survival analysis of cancer hallmark genes. Sci Rep. 2021;11:6047. 33723286 10.1038/s41598-021-84787-5 PMC7961001

[31] Tang Z, Li C, Kang B, Gao G, Li C, Zhang Z. GEPIA: a web server for cancer and normal gene expression profiling and interactive analyses. Nucleic Acids Res. 2017;45:W98–102. 28407145 10.1093/nar/gkx247 PMC5570223

[32] Zhang J, Luo A, Huang F, Gong T, Liu Z. SERPINE2 promotes esophageal squamous cell carcinoma metastasis by activating BMP4. Cancer Lett. 2020;469:390–8. 31730904 10.1016/j.canlet.2019.11.011

[33] Wagenblast E, Soto M, Gutiérrez-Ángel S, Hartl CA, Gable AL, Maceli AR, et al. A model of breast cancer heterogeneity reveals vascular mimicry as a driver of metastasis. Nature. 2015;520:358–62. 25855289 10.1038/nature14403 PMC4634366

[34] Monard D. SERPINE2/Protease Nexin-1 in vivo multiple functions: does the puzzle make sense? Semin Cell Dev Biol. 2017;62:160–9. 27545616 10.1016/j.semcdb.2016.08.012

[35] Zhang J, Wu Q, Zhu L, Xie S, Tu L, Yang Y, et al. SERPINE2/PN-1 regulates the DNA damage response and radioresistance by activating ATM in lung cancer. Cancer Lett. 2022;524:268–83. 34648881 10.1016/j.canlet.2021.10.001

[36] Bergeron S, Lemieux E, Durand V, Cagnol S, Carrier JC, Lussier JG, et al. The serine protease inhibitor serpinE2 is a novel target of ERK signaling involved in human colorectal tumorigenesis. Mol Cancer. 2010;9:271. 20942929 10.1186/1476-4598-9-271 PMC2967542

[37] Buchholz M, Biebl A, Neesse A, Wagner M, Iwamura T, Leder G, et al. SERPINE2 (protease nexin I) promotes extracellular matrix production and local invasion of pancreatic tumors in vivo. Cancer Res. 2003;63:4945–51. 12941819

[38] Fayard B, Bianchi F, Dey J, Moreno E, Djaffer S, Hynes NE, et al. The serine protease inhibitor protease nexin-1 controls mammary cancer metastasis through LRP-1-mediated MMP-9 expression. Cancer Res. 2009;69:5690–8. 19584287 10.1158/0008-5472.CAN-08-4573

[39] Azizi E, Carr AJ, Plitas G, Cornish AE, Konopacki C, Prabhakaran S, et al. Single-cell map of diverse immune phenotypes in the breast tumor microenvironment. Cell. 2018;174:1293–308.e36. 29961579 10.1016/j.cell.2018.05.060 PMC6348010

[40] Nam AS, Chaligne R, Landau DA. Integrating genetic and non-genetic determinants of cancer evolution by single-cell multi-omics. Nat Rev Genet. 2021;22:3–18. 32807900 10.1038/s41576-020-0265-5 PMC8450921

[41] Chen B, Scurrah CR, McKinley ET, Simmons AJ, Ramirez-Solano MA, Zhu X, et al. Differential pre-malignant programs and microenvironment chart distinct paths to malignancy in human colorectal polyps. Cell. 2021;184:6262–80.e26. 34910928 10.1016/j.cell.2021.11.031 PMC8941949

[42] Jerby-Arnon L, Shah P, Cuoco MS, Rodman C, Su M-J, Melms JC, et al. A cancer cell program promotes T cell exclusion and resistance to checkpoint blockade. Cell. 2018;175:984–97.e24. 30388455 10.1016/j.cell.2018.09.006 PMC6410377

[43] Maynard A, McCoach CE, Rotow JK, Harris L, Haderk F, Kerr DL, et al. Therapy-induced evolution of human lung cancer revealed by single-cell RNA sequencing. Cell. 2020;182:1232–51.e22. 32822576 10.1016/j.cell.2020.07.017 PMC7484178

[44] Helmink BA, Reddy SM, Gao J, Zhang S, Basar R, Thakur R, et al. B cells and tertiary lymphoid structures promote immunotherapy response. Nature. 2020;577:549–55. 31942075 10.1038/s41586-019-1922-8 PMC8762581

[45] Pastushenko I, Blanpain C. EMT transition states during tumor progression and metastasis. Trends Cell Biol. 2019;29:212–26. 30594349 10.1016/j.tcb.2018.12.001

[46] Serrano-Gomez SJ, Maziveyi M, Alahari SK. Regulation of epithelial-mesenchymal transition through epigenetic and post-translational modifications. Mol Cancer. 2016;15:18. 26905733 10.1186/s12943-016-0502-x PMC4765192

[47] Wu QW. Serpine2, a potential novel target for combating melanoma metastasis. Am J Transl Res. 2016;8:1985–97. 27347308 PMC4891413

[48] Bakir B, Chiarella AM, Pitarresi JR, Rustgi AK. EMT, MET, plasticity, and tumor metastasis. Trends Cell Biol. 2020;30:764–76. 32800658 10.1016/j.tcb.2020.07.003 PMC7647095

[49] Lambert AW, Pattabiraman DR, Weinberg RA. Emerging biological principles of metastasis. Cell. 2017;168:670–91. 28187288 10.1016/j.cell.2016.11.037 PMC5308465

[50] Smirnova T, Bonapace L, MacDonald G, Kondo S, Wyckoff J, Ebersbach H, et al. Serpin E2 promotes breast cancer metastasis by remodeling the tumor matrix and polarizing tumor associated macrophages. Oncotarget. 2016;7:82289–304. 27793045 10.18632/oncotarget.12927 PMC5347692

[51] Vaillant C, Valdivieso P, Nuciforo S, Kool M, Schwarzentruber-Schauerte A, Méreau H, et al. Serpine2/PN-1 is required for proliferative expansion of pre-neoplastic lesions and malignant progression to medulloblastoma. PLoS ONE. 2015;10:e0124870. 25901736 10.1371/journal.pone.0124870 PMC4406471

[52] Launay S, Maubert E, Lebeurrier N, Tennstaedt A, Campioni M, Docagne F, et al. HtrA1-dependent proteolysis of TGF-beta controls both neuronal maturation and developmental survival. Cell Death Differ. 2008;15:1408–16. 18551132 10.1038/cdd.2008.82

