# Correction: Single-cell RNA-seq integrated with multi-omics reveals SERPINE2 as a target for metastasis in advanced renal cell carcinoma

**DOI:** 10.1038/s41419-026-08472-z

**Published:** 2026-03-24

**Authors:** Wen-jin Chen, Ke-qin Dong, Xiu-wu Pan, Si-shun Gan, Da Xu, Jia-xin Chen, Wei-jie Chen, Wen-yan Li, Yu-qi Wang, Wang Zhou, Brian Rini, Xin-gang Cui

**Affiliations:** 1 Department of Urology, Xinhua Hospital, School of Medicine, Shanghai Jiaotong University, 1665 Kongjiang Road, Shanghai, 200092 China; 2 Department of Urology, Third Affiliated Hospital of the Second Military Medical University, Shanghai, 201805 China; 3 Department of Urology, General Hospital of Central Theater Command of PLA, Wuhan, 430070 China; 4 Division of Hematology Oncology, Vanderbilt University Medical Center, Nashville, TN USA

Correction to: *Cell Death & Disease* 10.1038/s41419-023-05566-w, published online 22 March 2022

During a careful post-publication review, we noticed an error in Figure S6F (the supplementary figure) where the images for the Day 28 and Day 56 in the Vector-SERPINE2 group were inadvertently duplicated.

We also unfortunately found a mistake about the vertical axis in Figure 6C.

We have referred back to the original data, and realized that inadvertent oversights occurred during the figure assembly process and were not detected prior to publication. We would like to emphasize that this correction does not affect the results, interpretation, or conclusions of the study.

**Incorrect Figure 6C** 
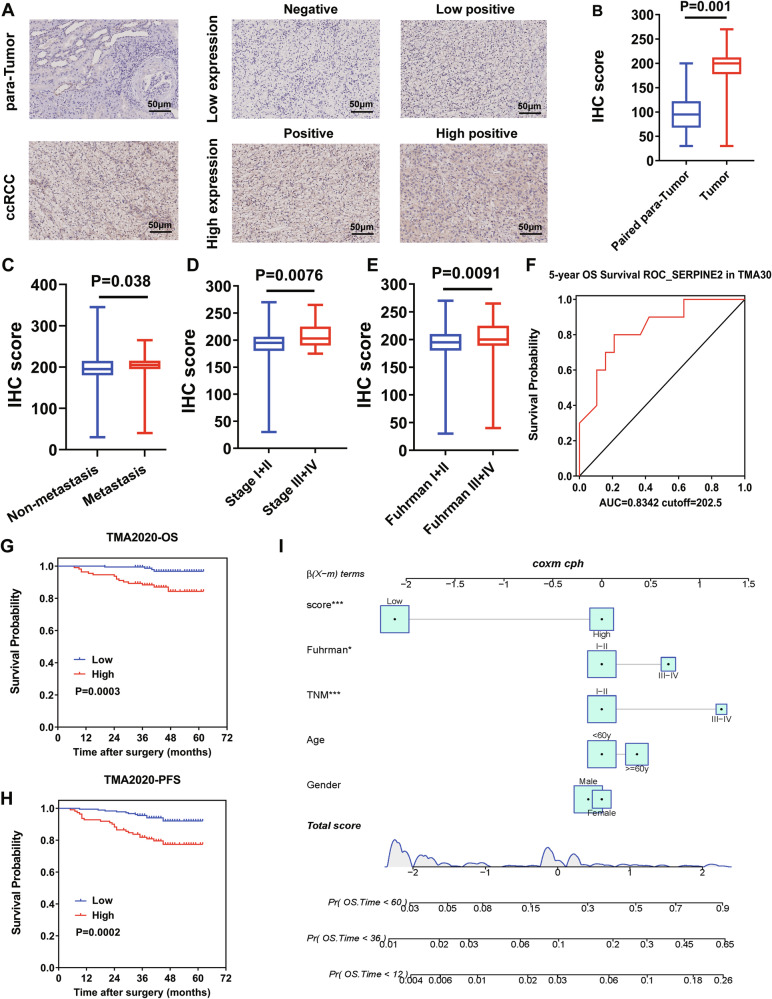


**Correct Figure 6C** 
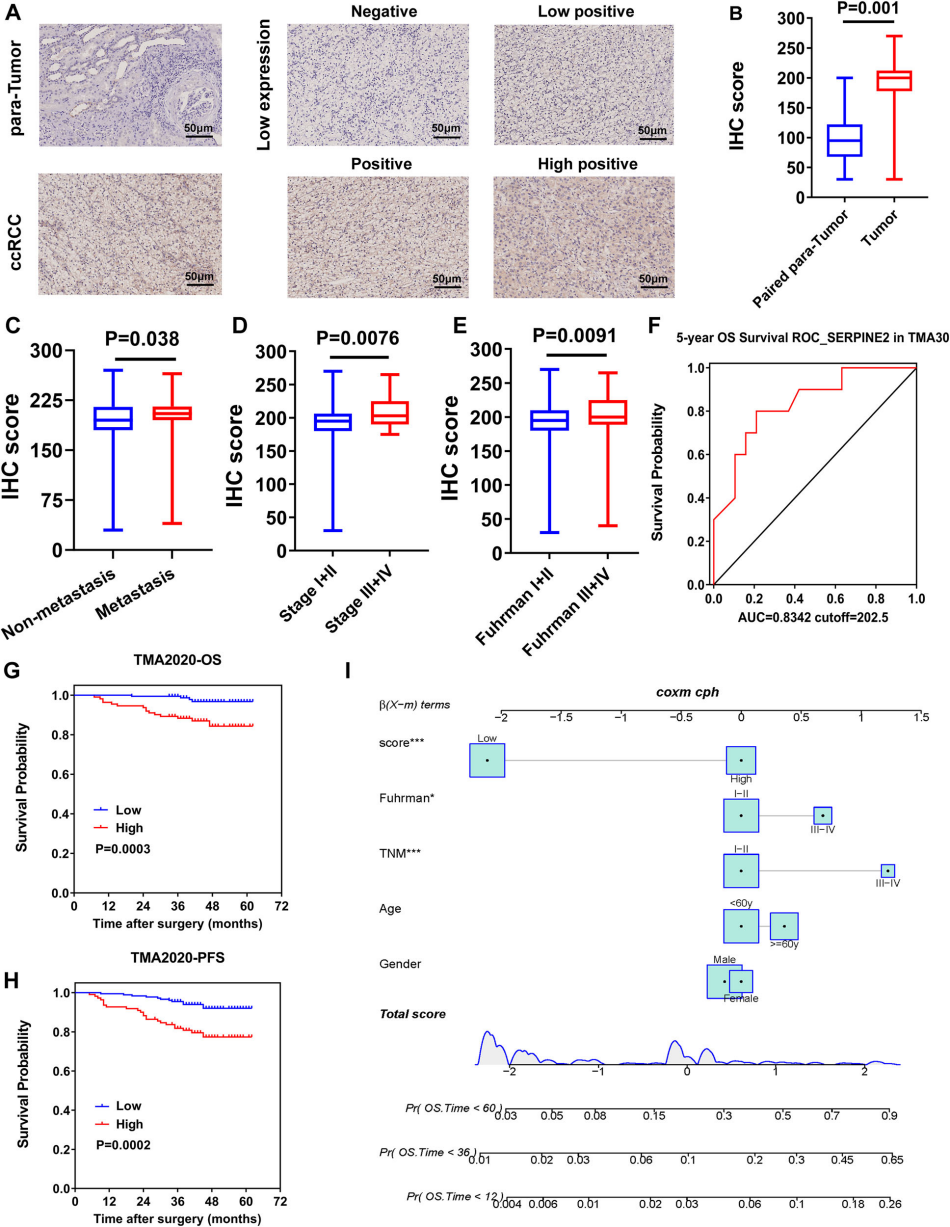


**Incorrect Figure S6F** 
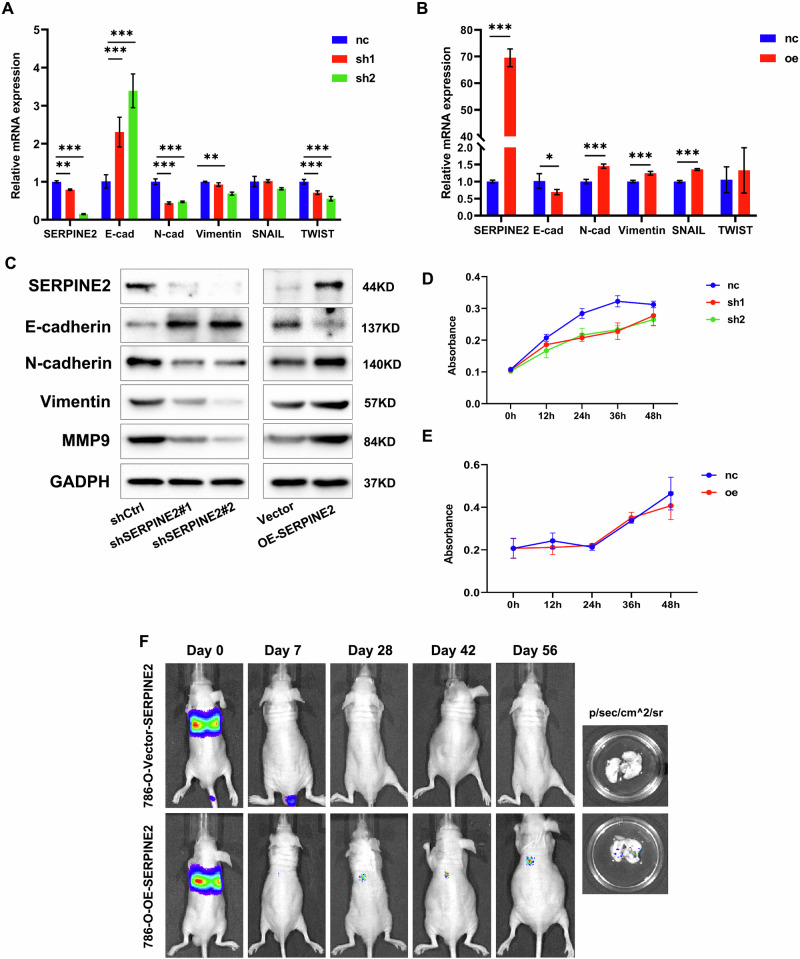


**Correct Figure S6F** 
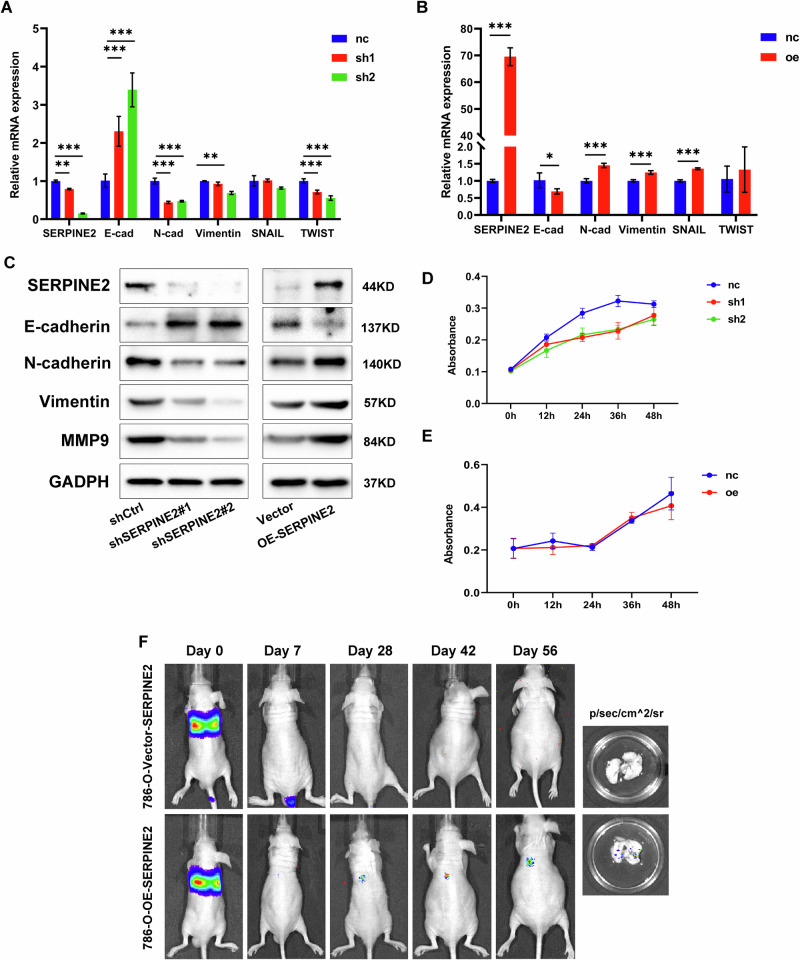


The original article has been corrected.

