# Supplementary material for: Single-cell RNA-seq integrated with multi-omics reveals SERPINE2 as a target for metastasis in advanced renal cell carcinoma
Source: Cell Death Dis. 2023 Jan 16;14(1):30. doi: 10.1038/s41419-023-05566-w (PMC9842647; doi:10.1038/s41419-023-05566-w)
Supplement: Supplementary file 1 — Supplementary figures [file 41419_2023_5566_MOESM1_ESM.pdf]

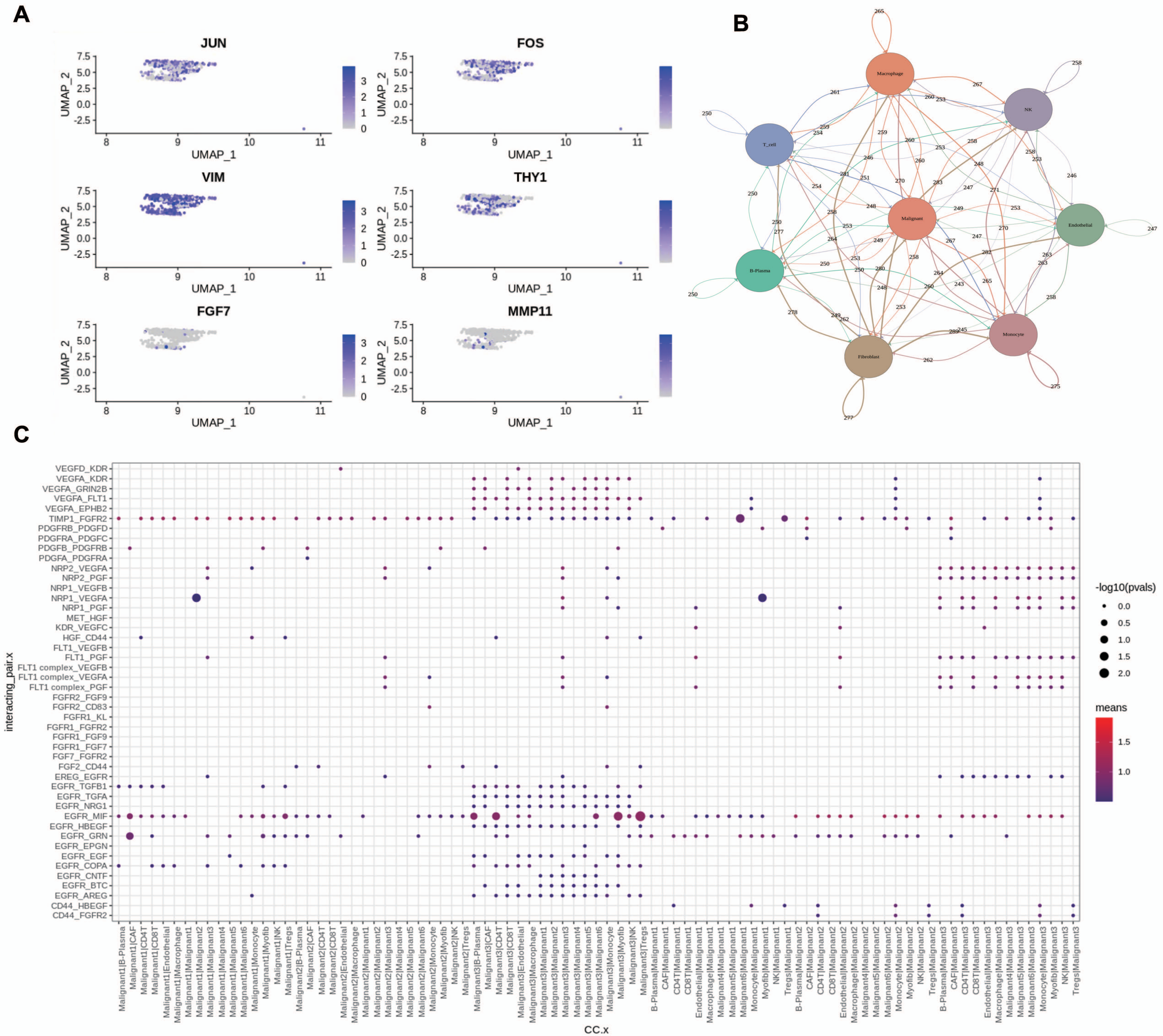
A
JUN
FOS
VIM
THY1
FGF7
MMP11
UMAP_1
UMAP_2
B
Macrophage
NK
T_cell
Malignant
Endothelial
B-Plasma
Monocyte
Fibroblast
C
interacting_pair.x
CC.x
-log10(pvals)
means

Fig.S1

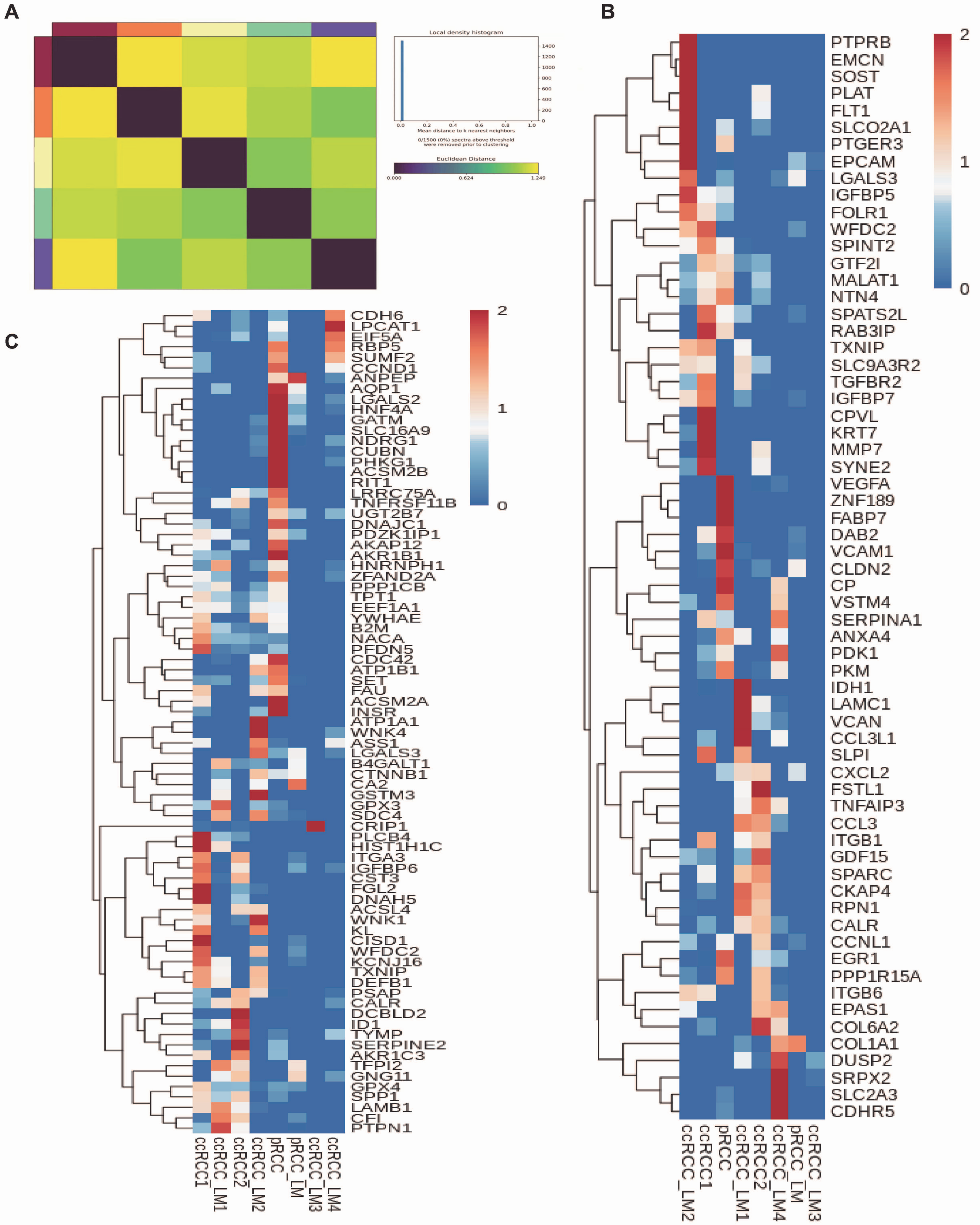

A
Local density histogram
1400
1200
1000
800
600
400
200
0
0.0
0.2
0.4
0.6
0.8
1.0
Mean distance to k nearest neighbors
0/1500 (0%) spectra above threshold were removed prior to clustering
Euclidean Distance
0.000
0.624
1.249
B
PTPRB
EMCN
SOST
PLAT
FLT1
SLCO2A1
PTGER3
EPCAM
LGALS3
IGFBP5
FOLR1
WFDC2
SPINT2
GTF2I
MALAT1
NTN4
SPATS2L
RAB3IP
TXNIP
SLC9A3R2
TGFBR2
IGFBP7
CPVL
KRT7
MMP7
SYNE2
VEGFA
ZNF189
FABP7
DAB2
VCAM1
CLDN2
CP
VSTM4
SERPINA1
ANXA4
PDK1
PKM
IDH1
LAMC1
VCAN
CCL3L1
SLPI
CXCL2
FSTL1
TNFAIP3
CCL3
ITGB1
GDF15
SPARC
CKAP4
RPN1
CALR
CCNL1
EGR1
PPP1R15A
ITGB6
EPAS1
COL6A2
COL1A1
DUSP2
SRPX2
SLC2A3
CDHR5
2
1
0
ccRCC_LM2
ccRCC1
pRCC
ccRCC_LM1
ccRCC2
ccRCC_LM4
pRCC_LM
ccRCC_LM3
C
CDH6
LPCAT1
EIF5A
RBP5
SUMF2
CCND1
ANPEP
AQP1
LGALS2
HNF4A
GATM
SLC16A9
NDRG1
CUBN
PHKG1
ACSM2B
RIT1
LRRC75A
TNFRSF11B
UGT2B7
DNAJC1
PDZK1IP1
AKAP12
AKR1B1
HNRNPH1
ZFAND2A
PPP1CB
TPT1
EEF1A1
YWHAE
B2M
NACA
PFDN5
CDC42
ATP1B1
SET
FAU
ACSM2A
INSR
ATP1A1
WNK4
ASS1
LGALS3
B4GALT1
CTNNB1
CA2
GSTM3
GPX3
SDC4
CRIP1
PLCB4
HIST1H1C
ITGA3
IGFBP6
CST3
FGL2
DNAH5
ACSL4
WNK1
KL
CISD1
WFDC2
KCNJ16
TXNIP
DEFB1
PSAP
CALR
DCBLD2
ID1
TYMP
SERPINE2
AKR1C3
TFPI2
GNG11
GPX4
SPP1
LAMB1
CFI
PTPN1
2
1
0
ccRCC1
ccRCC_LM1
ccRCC2
ccRCC_LM2
pRCC
pRCC_LM
ccRCC_LM3
ccRCC_LM4

Fig.S2

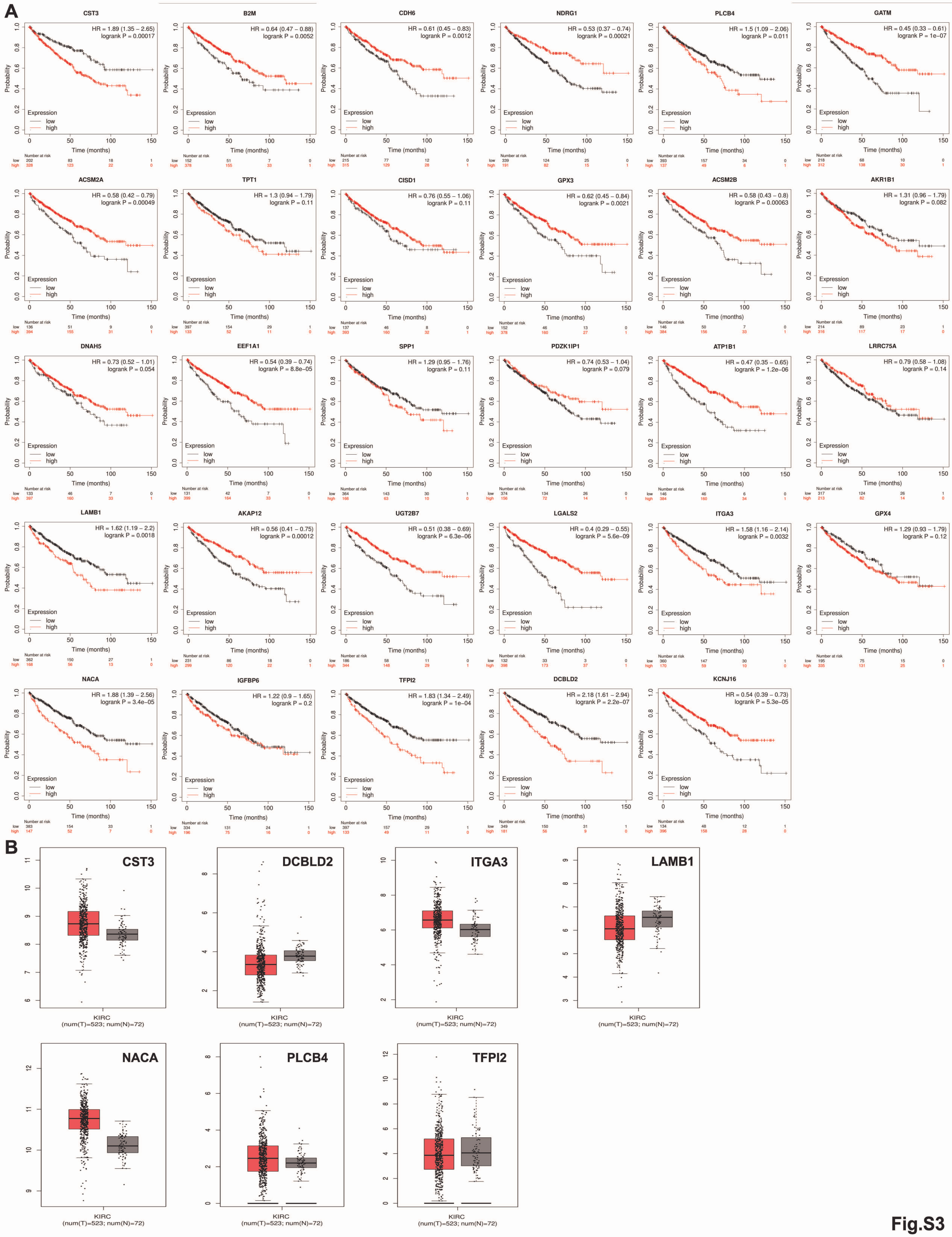
A
CST3
HR = 1.89 (1.35 – 2.65)
logrank P = 0.00017
B2M
HR = 0.64 (0.47 – 0.88)
logrank P = 0.0052
CDH6
HR = 0.61 (0.45 – 0.83)
logrank P = 0.0012
NDRG1
HR = 0.53 (0.37 – 0.74)
logrank P = 0.00021
PLCB4
HR = 1.5 (1.09 – 2.06)
logrank P = 0.011
GATM
HR = 0.45 (0.33 – 0.61)
logrank P = 1e−07
ACSM2A
HR = 0.58 (0.42 – 0.79)
logrank P = 0.00049
TPT1
HR = 1.3 (0.94 – 1.79)
logrank P = 0.11
CISD1
HR = 0.76 (0.55 – 1.06)
logrank P = 0.11
GPX3
HR = 0.62 (0.45 – 0.84)
logrank P = 0.0021
ACSM2B
HR = 0.58 (0.43 – 0.8)
logrank P = 0.00063
AKR1B1
HR = 1.31 (0.96 – 1.79)
logrank P = 0.082
DNAH5
HR = 0.73 (0.52 – 1.01)
logrank P = 0.054
EEF1A1
HR = 0.54 (0.39 – 0.74)
logrank P = 8.8e−05
SPP1
HR = 1.29 (0.95 – 1.76)
logrank P = 0.11
PDZK1IP1
HR = 0.74 (0.53 – 1.04)
logrank P = 0.079
ATP1B1
HR = 0.47 (0.35 – 0.65)
logrank P = 1.2e−06
LRRC75A
HR = 0.79 (0.58 – 1.08)
logrank P = 0.14
LAMB1
HR = 1.62 (1.19 – 2.2)
logrank P = 0.0018
AKAP12
HR = 0.56 (0.41 – 0.75)
logrank P = 0.00012
UGT2B7
HR = 0.51 (0.38 – 0.69)
logrank P = 6.3e−06
LGALS2
HR = 0.4 (0.29 – 0.55)
logrank P = 5.6e−09
ITGA3
HR = 1.58 (1.16 – 2.14)
logrank P = 0.0032
GPX4
HR = 1.29 (0.93 – 1.79)
logrank P = 0.12
NACA
HR = 1.88 (1.39 – 2.56)
logrank P = 3.4e−05
IGFBP6
HR = 1.22 (0.9 – 1.65)
logrank P = 0.2
TFPI2
HR = 1.83 (1.34 – 2.49)
logrank P = 1e−04
DCBLD2
HR = 2.18 (1.61 – 2.94)
logrank P = 2.2e−07
KCNJ16
HR = 0.54 (0.39 – 0.73)
logrank P = 5.3e−05
Probability
Time (months)
Expression
low
high
Number at risk
B
CST3
DCBLD2
ITGA3
LAMB1
NACA
PLCB4
TFPI2
KIRC
(num(T)=523; num(N)=72)

Fig.S3

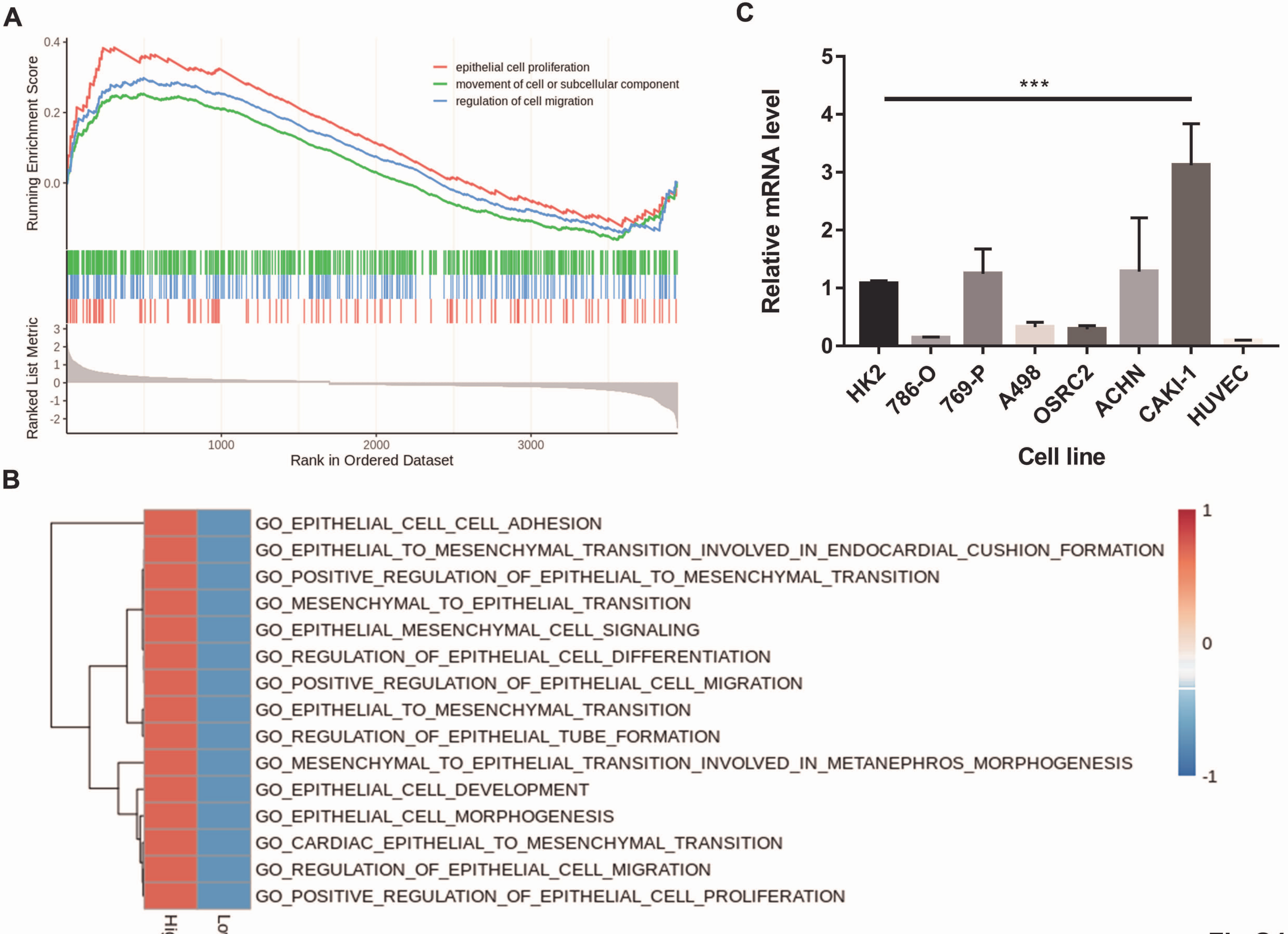
A
Running Enrichment Score
0.4
0.2
0.0
epithelial cell proliferation
movement of cell or subcellular component
regulation of cell migration
Ranked List Metric
3
2
1
0
-1
-2
1000
2000
3000
Rank in Ordered Dataset
C
Relative mRNA level
5
4
3
2
1
0
***
HK2
786-O
769-P
A498
OSRC2
ACHN
CAKI-1
HUVEC
Cell line
B
GO_EPITHELIAL_CELL_CELL_ADHESION
GO_EPITHELIAL_TO_MESENCHYMAL_TRANSITION_INVOLVED_IN_ENDOCARDIAL_CUSHION_FORMATION
GO_POSITIVE_REGULATION_OF_EPITHELIAL_TO_MESENCHYMAL_TRANSITION
GO_MESENCHYMAL_TO_EPITHELIAL_TRANSITION
GO_EPITHELIAL_MESENCHYMAL_CELL_SIGNALING
GO_REGULATION_OF_EPITHELIAL_CELL_DIFFERENTIATION
GO_POSITIVE_REGULATION_OF_EPITHELIAL_CELL_MIGRATION
GO_EPITHELIAL_TO_MESENCHYMAL_TRANSITION
GO_REGULATION_OF_EPITHELIAL_TUBE_FORMATION
GO_MESENCHYMAL_TO_EPITHELIAL_TRANSITION_INVOLVED_IN_METANEPHROS_MORPHOGENESIS
GO_EPITHELIAL_CELL_DEVELOPMENT
GO_EPITHELIAL_CELL_MORPHOGENESIS
GO_CARDIAC_EPITHELIAL_TO_MESENCHYMAL_TRANSITION
GO_REGULATION_OF_EPITHELIAL_CELL_MIGRATION
GO_POSITIVE_REGULATION_OF_EPITHELIAL_CELL_PROLIFERATION
1
0
-1
High
Low

Fig.S4

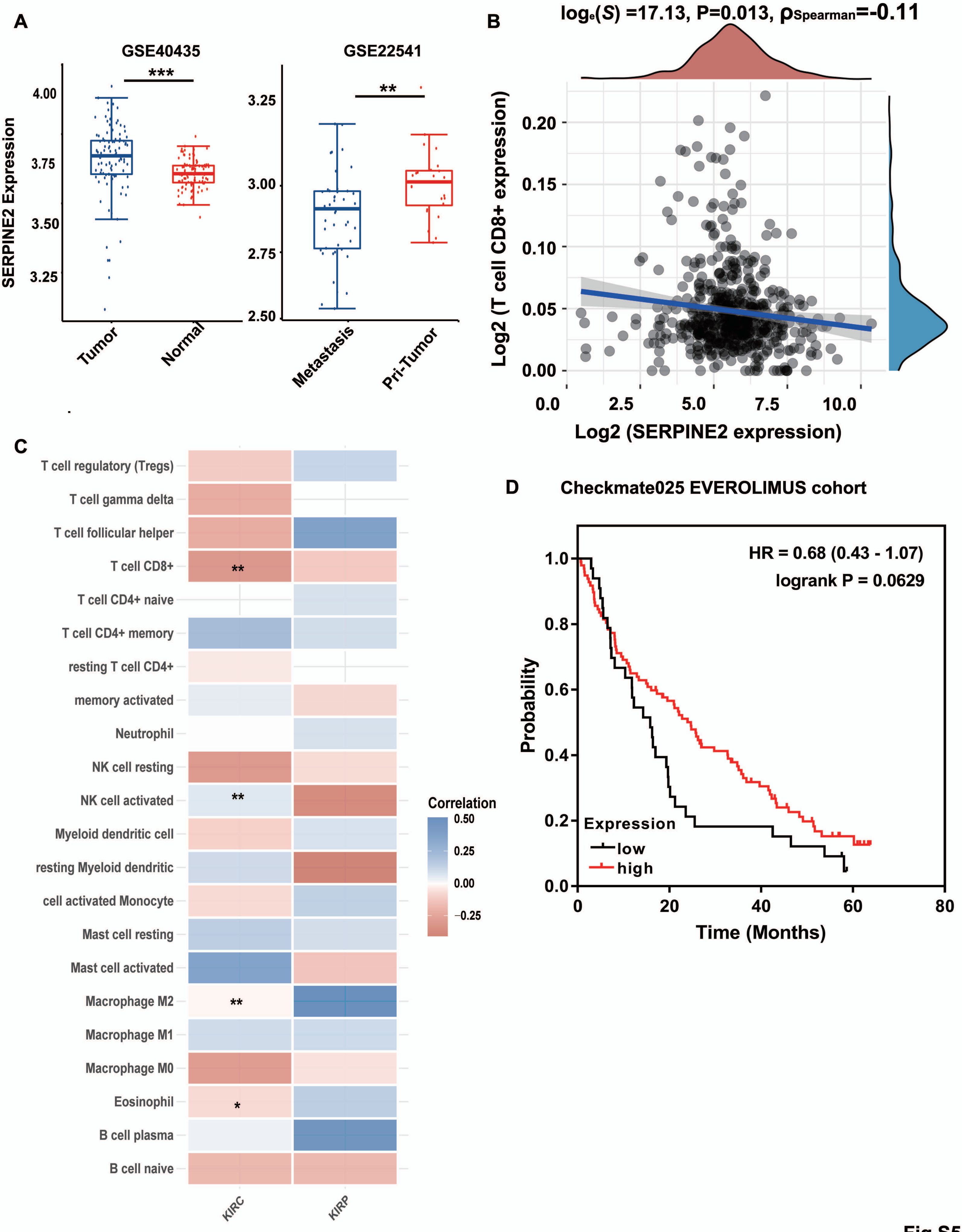
A
GSE40435
***
4.00
3.75
3.50
3.25
SERPINE2 Expression
Tumor
Normal
GSE22541
**
3.25
3.00
2.75
2.50
Metastasis
Pri-Tumor
B
loge(S) =17.13, P=0.013, ρSpearman=-0.11
Log2 (T cell CD8+ expression)
0.20
0.15
0.10
0.05
0.00
0.0
2.5
5.0
7.5
10.0
Log2 (SERPINE2 expression)
C
T cell regulatory (Tregs)
T cell gamma delta
T cell follicular helper
T cell CD8+
**
T cell CD4+ naive
T cell CD4+ memory
resting T cell CD4+
memory activated
Neutrophil
NK cell resting
NK cell activated
**
Myeloid dendritic cell
resting Myeloid dendritic
cell activated Monocyte
Mast cell resting
Mast cell activated
Macrophage M2
**
Macrophage M1
Macrophage M0
Eosinophil
*
B cell plasma
B cell naive
KIRC
KIRP
Correlation
0.50
0.25
0.00
−0.25
D
Checkmate025 EVEROLIMUS cohort
HR = 0.68 (0.43 - 1.07)
logrank P = 0.0629
Probability
1.0
0.8
0.6
0.4
0.2
0.0
Expression
low
high
0
20
40
60
80
Time (Months)

Fig.S5

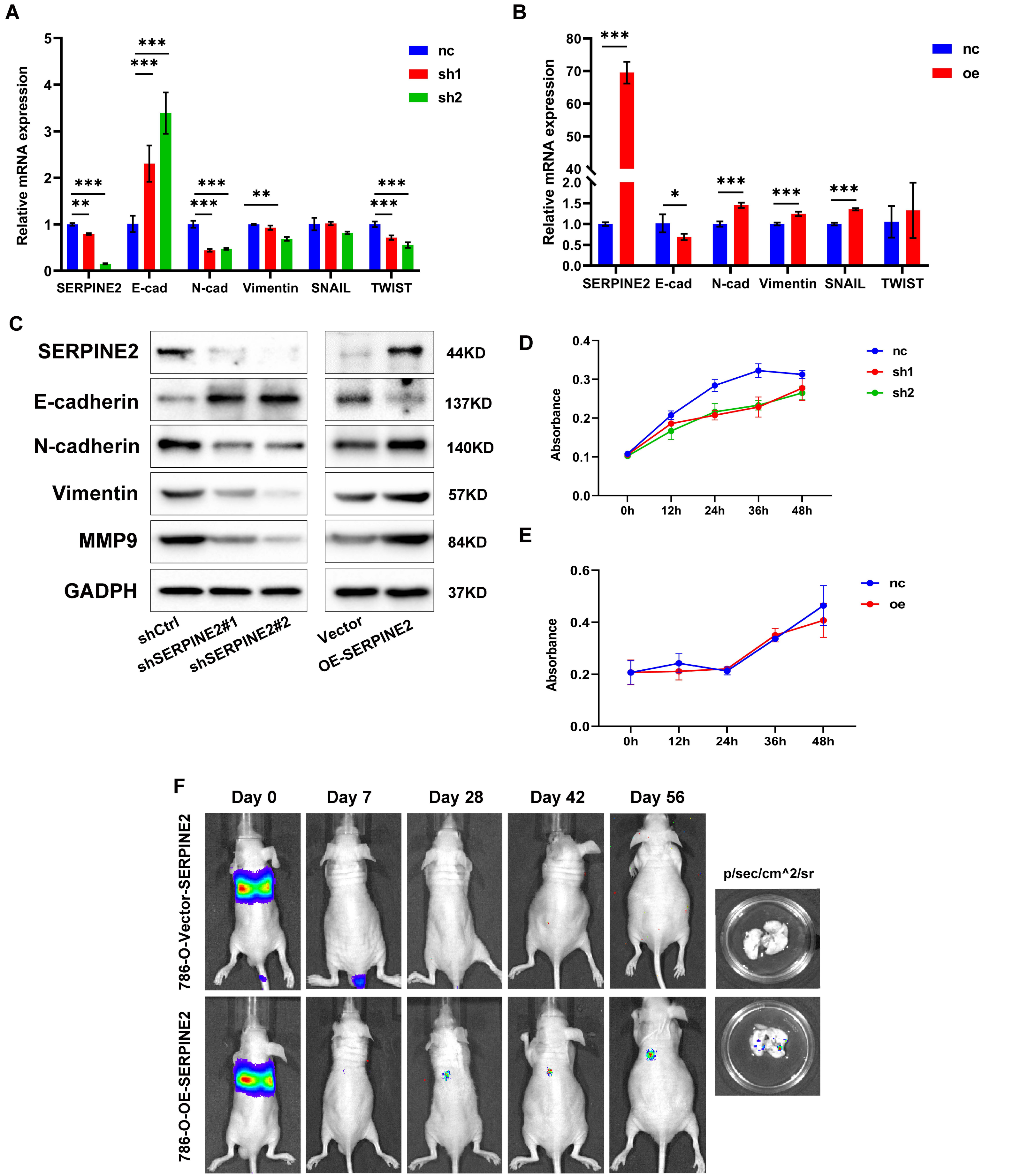
A
Relative mRNA expression
nc
sh1
sh2
SERPINE2
E-cad
N-cad
Vimentin
SNAIL
TWIST
B
Relative mRNA expression
nc
oe
SERPINE2
E-cad
N-cad
Vimentin
SNAIL
TWIST
C
SERPINE2
E-cadherin
N-cadherin
Vimentin
MMP9
GADPH
44KD
137KD
140KD
57KD
84KD
37KD
shCtrl
shSERPINE2#1
shSERPINE2#2
Vector
OE-SERPINE2
D
Absorbance
nc
sh1
sh2
0h
12h
24h
36h
48h
E
Absorbance
nc
oe
0h
12h
24h
36h
48h
F
Day 0
Day 7
Day 28
Day 42
Day 56
786-O-Vector-SERPINE2
786-O-OE-SERPINE2
p/sec/cm^2/sr

Fig.S6

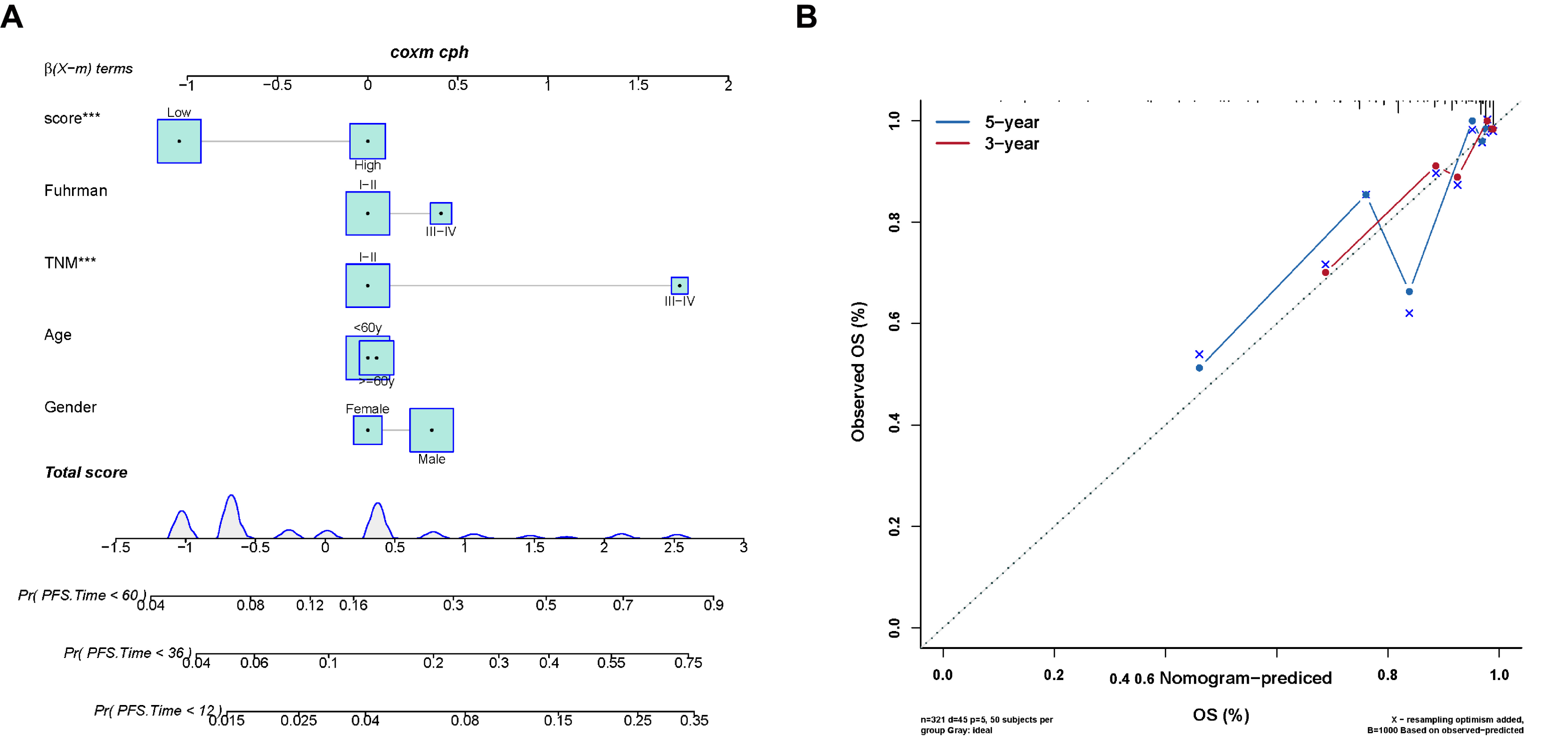

A
coxm cph
β(X−m) terms
score***
Low
High
Fuhrman
I−II
III−IV
TNM***
I−II
III−IV
Age
<60y
>=60y
Gender
Female
Male
Total score
Pr(PFS.Time < 60)
Pr(PFS.Time < 36)
Pr(PFS.Time < 12)
B
5−year
3−year
Observed OS (%)
Nomogram−prediced OS (%)
n=321 d=45 p=5, 50 subjects per group Gray: ideal
X − resampling optimism added, B=1000 Based on observed−predicted

Fig.S7
