# Supplementary material for: Single-cell RNA-seq integrated with multi-omics reveals SERPINE2 as a target for metastasis in advanced renal cell carcinoma
Source: Cell Death Dis. 2023 Jan 16;14(1):30. doi: 10.1038/s41419-023-05566-w (PMC9842647; doi:10.1038/s41419-023-05566-w)
Supplement: Supplementary file 2 — Supplementary figure legends [file 41419_2023_5566_MOESM2_ESM.docx]

**Supplementary figure legends**

Fig. S1. (A) The expression of JUN, FOS, VIM, THY1, FGF7, MMP11 in Fibroblasts. (B) iTALK results revealed the interactions between the cell clusters. Each circle represents a cluster; the arrows represent the interactions between circles; the number on the arrows represent the number of the interactions. (C) The interaction network established by CellphoneDB; size and number of lines represents interaction counts. Row, the pairs of ligands and receptors; Column, the cell-cell contact.

Fig. S2. (A) The correlation heatmap to choose the number of optimal NMF cluster. (B) The heatmap shows genes expression in the Metastasis I program of each tumor sample. (C) The heatmap shows genes expression in the Metastasis II program of each tumor sample.

Fig. S3. (A) The KM survival curve of each Venn gene in the TCGA database. (B) Boxplot shows the expression between tumor and normal tissue for the gene with poorer OS in the high-expression group of TCGA.

Fig. S4. (A) The GSEA result showed the epithelial invasion pathways were enriched in the cells with higher SERPINE2 expression. (B) The GSVA heatmap shows the pathways enriched between SERPINE2 high and low cells. (C) The qPCR result shows the relative mRNA expression of SERPINE2 in each celline, compared to HK-2, the normal epithelial cell.

Fig. S5. (A) Left, The SERPINE2 expression between tumors (n=101) and normal tissues (n=101) in GSE40435. Right, The SERPINE2 expression among primary (n=44) and metastatic tumors (n=24) in GSE22541. Data was obtained from https://www.aclbi.com/static/index.html#/geo. (B) The correlation between the SERPINE2 expression and T cell CD8+ expression in TCGA. (C) The cibersorts result shows the correlation between the SERPINE2 high/low expression and immune cells abundance. (D) The KM survival curve of SERPINE2-high and SERPINE2-low group in the cohort of clinical trials Checkmate025 with Everolimus.

Fig. S6. (A) The qPCR results show EMT markers expressions among shCtrl, shSERPINE2#1 and shSERPINE2#2 group in Caki-1 cells, relative change compared with shCtrl group. (B) The qPCR results show EMT markers expressions between Vector, OE-SERPINE2 group in 786-O cell, relative change compared with Vector group. (C) The western blot results (D) The CCK-8 result shows the proliferation among shCtrl, shSERPINE2#1 and shSERPINE2#2 group in Caki-1 cells. (E) The CCK-8 result shows the proliferation between Vector, OE-SERPINE2 group in 786-O cell. (F) Images of luciferase intensity and lung metastases are presented. Photon flux levels were examined in the 786-O-OE-SERPINE2 or 786-O-NC-SERPINE2 of mice, and the results are presented as the level in lung metastases.

Fig. S7. (A) The nomogram for 1-year, 3-year, and 5-year PFS in our cohort. (B) The calibration plot of nomogram for 1-year, 3-year, and 5-year OS.
