# Supplementary figures and images for: Single-cell RNA-seq integrated with multi-omics reveals SERPINE2 as a target for metastasis in advanced renal cell carcinoma
Source: Cell Death Dis. 2023 Jan 16;14(1):30. doi: 10.1038/s41419-023-05566-w (PMC9842647; doi:10.1038/s41419-023-05566-w)

Sh-NC    Sh-2    Sh-3    OE-NC    OE-SERPINE2

SERPINE2

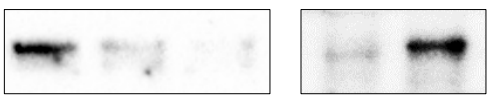

E-cadherin

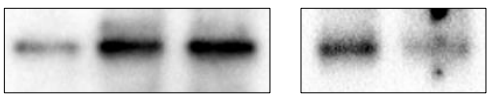

N-cadherin

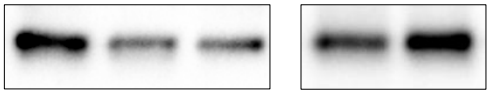

Vimentin

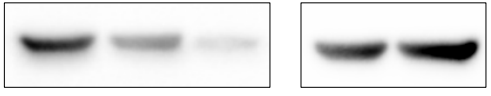

MMP9

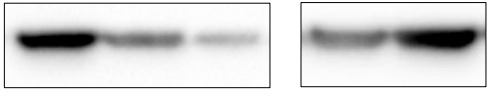

GAPDH

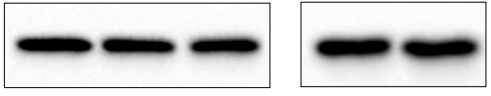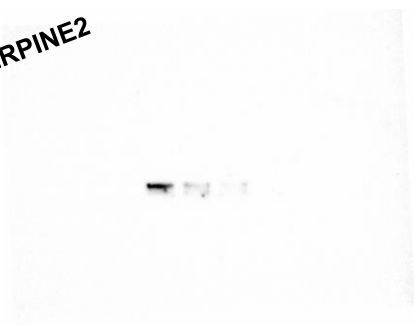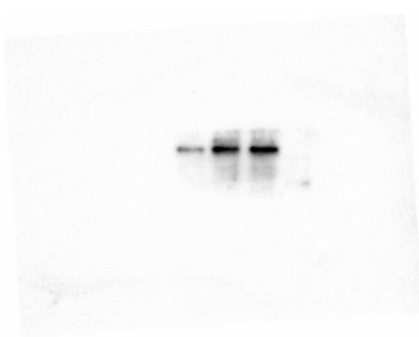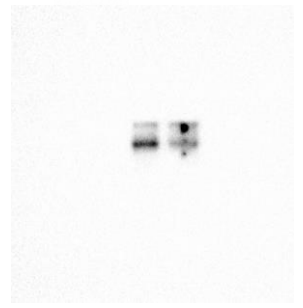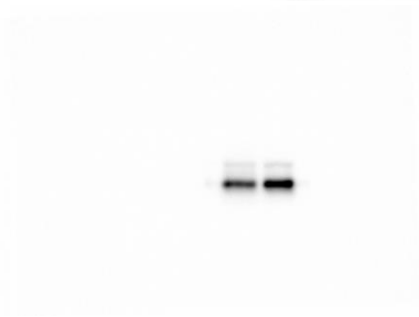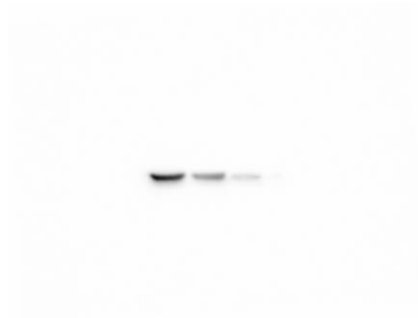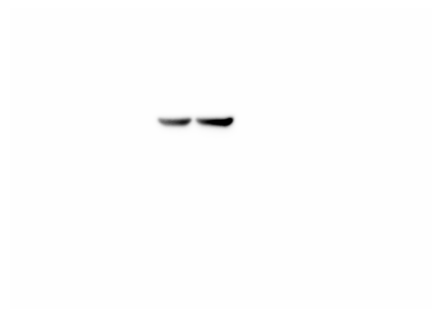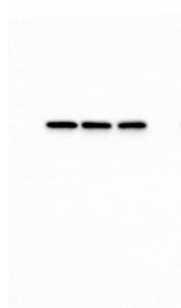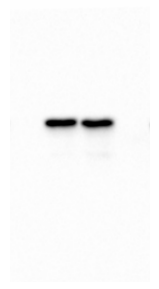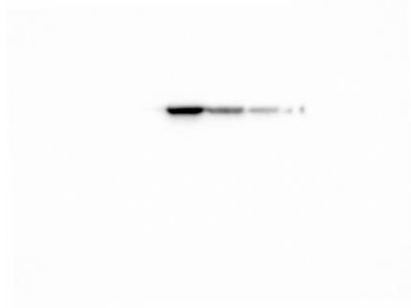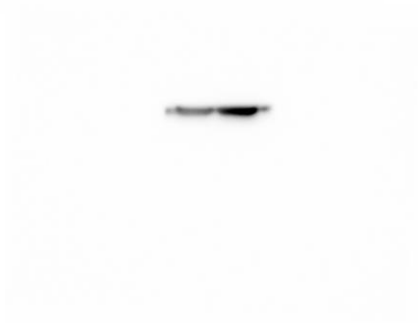

Supplement: Supplementary file 13 — Extended Data [file 41419_2023_5566_MOESM13_ESM.pdf]
